# Targeted Deletion of Peroxiredoxin 1 Enhances Anti‐Tumor Immunity in Colorectal Cancer by Reprogramming the Immunosuppressive Tumor‐Associated Macrophages

**DOI:** 10.1002/mco2.70495

**Published:** 2025-11-24

**Authors:** Yuqi Sun, Jinli Han, Nianhua Yu, Jinglin Qin, Xiaohui Wang, Xi Li, Yujia Song, Xiaoxue Xu, Xinfeng Yu

**Affiliations:** ^1^ Department of Pharmacology, School of Basic Medical Sciences Capital Medical University Beijing China; ^2^ Department of Neurobiology, School of Basic Medical Sciences, Beijing Key Laboratory of Neural Regeneration and Repair Capital Medical University Beijing China; ^3^ Department of General Surgery Xuanwu Hospital Capital Medical University Beijing China; ^4^ Department of Core Facility Center Capital Medical University Beijing China

**Keywords:** colorectal cancer, immune suppression, macrophage polarization, PRDX1

## Abstract

Peroxiredoxin 1 (PRDX1) overexpression in colorectal cancer (CRC) correlates with poor prognosis and reduced T‐cell infiltration. However, the mechanism underlying PRDX1‐mediated immune suppression remains elusive. In this study, we found that knockout of PRDX1 robustly suppressed AOM/DSS‐induced colonic adenocarcinoma compared with wild‐type C57BL/6J mice, accompanied by highly infiltrated CD4^+^/CD8^+ ^T cells and reduced CD163^+^ tumor‐associated macrophages (TAMs). Furthermore, PRDX1 knockdown in CRC cells inhibited M2 macrophage polarization by impairing hypoxia‐inducible factor 1α (HIF‐1α)/GLUT‐1‐mediated glycolysis and lactate secretion. Mechanistically, PRDX1 binds to Cullin‐2 as a molecular chaperone, thereby suppressing ubiquitination and degradation of HIF‐1α. The PRDX1^Cys83Ser^ mutant abolished the ability to bind to Cullin‐2, suggesting that Cys83 is an active site of PRDX1 in regulating HIF‐1α/GLUT‐1‐mediated glycolysis. Importantly, PRDX1 deletion in macrophages reversed the immunosuppressive phenotype and reciprocally enhanced the phagocytosis, inhibited CRC cell growth and migration. Cytokine assay demonstrated that PRDX1 deficiency increased IL‐1β and TNF‐α secretion by activating the JAK/STAT1/NF‐κB pathway, promoting M1 macrophage polarization. Notably, PRDX1 knockout macrophages inhibited syngeneic tumor growth and enhanced sensitivity to anti‐PD‐1 therapy in vivo. In conclusion, targeted deletion of PRDX1 enhances anti‐tumor immunity in CRC by reprogramming the immunosuppressive TAMs, revealing a novel role of PRDX1 as a potential drug target during anti‐tumor immunotherapy.

## Graphical abstract

This study reveals a dual mechanism by which PRDX1 knockdown alleviates immunosuppression in CRC. On the one hand, PRDX1 knockdown in CRC cells inhibits M2 macrophage polarization by destabilizing HIF‐1α through impaired binding to Cullin‐2, thereby suppressing GLUT‐1‐dependent glycolysis and lactate secretion. On the other hand, loss of PRDX1 in macrophages restrains M2 polarization and enhances T‐cell‐mediated anti‐tumor immunity by promoting the secretion of inflammatory factors (IL‐1β and TNFα) via activation of JAK‐STAT1/NF‐κB signaling pathways. These results underscore the therapeutic potential of targeting PRDX1 to reverse immunosuppression and enhance anti‐tumor immunity in CRC.

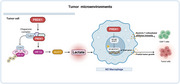

## 1 Introduction

Immune checkpoint inhibitors (ICIs) have emerged as a breakthrough in cancer therapy by activating the antitumor immunity, particularly in CRC with mismatch repair deficiency (dMMR) and/or high microsatellite instability (MSI‐H). Although PD‐L1/PD‐1 immunotherapy has shown efficacy, microsatellite stable (MSS) CRC patients account for more than 80% of metastatic CRC and show poor clinical response to ICIs monotherapy [1]. Among the immune‐infiltrating MSI‐H patients, approximately 30%–50% of metastatic CRC patients develop acquired drug resistance to immunotherapy [2]. Therefore, there remains an urgent need to explore the interplay between CRC cells and the tumor microenvironment (TME) to potentiate the anti‐tumor response to ICIs in CRC patients.

Accumulating evidence highlights immunosuppression in the TME as a critical barrier for achieving optimal clinical outcome in cancer immunotherapy [3]. Notably, the immunosuppressive TME is predominantly infiltrated by M2‐polarized tumor‐associated macrophages (TAMs), which have become a potential target for cancer immunotherapy. In cancers with ICIs therapy, PD‐L1 expression in antigen‐presenting cells, including TAMs, plays a pivotal role in orchestrating the immune response to PD‐1 blockade therapy [4]. Preclinical studies have demonstrated that depletion of macrophages significantly increases CD8^+^ T‐cell infiltration into tumor islets, thereby improving the efficacy of anti‐PD‐1/PD‐L1 treatment [5]. Macrophages possess remarkable functional plasticity and can be polarized into two major subsets. Classically activated (M1) macrophages are characterized by the production of pro‐inflammatory cytokines (IL‐1β and TNF‐ɑ) and exhibit anti‐cancer capabilities. Conversely, alternatively activated (M2) macrophages are educated by tumor cells to develop an immunosuppressive phenotype, facilitating tumor growth through the release of anti‐inflammatory factors (IL‐10 and TGF‐β) or direct suppression of CD8^+^ T‐cell cytotoxicity [6]. Therapeutic strategies targeting macrophages, including inhibition of macrophage recruitment, reprogramming of TAMs toward M1 polarization phenotype, or depletion of TAMs, have shown combinatorial effects with PD‐1/PD‐L1 blockade [7].

Reactive oxygen species (ROS) play a complex dual role in cancer biology. While excessive ROS can disrupt cellular redox homeostasis, leading to genomic instability and malignant transformation [8], they are also fundamental signaling molecules in immune cells. ROS play a fundamental role in innate and adaptive immunity by getting involved in phagocytosis, activation, and antigen presentation [9]. This has spurred interest in targeting ROS biology to enhance cancer immunotherapy. Peroxiredoxin 1 (PRDX1), a key antioxidant enzyme of the peroxiredoxin family, functions by eliminating excessive intracellular ROS and preventing oxidative stress‐mediated DNA damage. Beyond its well‐established antioxidant role, PRDX1 also exhibits distinct molecular chaperone activity that contributes to the regulation of protein stability [10]. We have found previously that PRDX1 is significantly upregulated in human CRC tissues and drives CRC progression by inhibiting ferroptosis via the molecular chaperone activity [11]. However, the impact of PRDX1 on the TME, particularly its potential role in anti‐tumor immunity, remained completely unexplored. The immunosuppressive TME is a major barrier to effective cancer therapy. A key immunosuppressive factor is lactate, the end‐product of glycolysis, which is abundantly secreted by metabolically active cancer cells. Excessive lactate modulates immune responses within the TME, promoting M2 polarization of TAMs via extracellular acidification [12, 13, 14].

This study aimed to investigate the role of PRDX1 in shaping the immunosuppressive TME. Our findings uncovered a novel role for PRDX1 in anti‐tumor immunity through the regulation of macrophage polarization. Mechanistically, knockdown of PRDX1 in CRC cells suppressed hypoxia‐inducible factor 1α (HIF‐1α)/GLUT‐1‐mediated glycolysis and lactate production, thereby inhibiting M2 polarization of macrophages. This effect results from the role of PRDX1 as a molecular chaperone for Cullin‐2. Reciprocally, PRDX1 deficiency in macrophages promotes M1 polarization, which significantly enhances T‐cell cytotoxicity and sensitizes to anti‐PD‐1 immunotherapy. Our findings reveal PRDX1 as a central regulator of the metabolic crosstalk between CRC cells and TAMs in TME, highlighting its potential as a novel therapeutic target to overcome immunosuppression in CRC.

## 2 Results

### 2.1 PRDX1 Deficiency Suppresses AOM/DSS‐Induced CRC and Infiltration of M2 Polarized TAMs in Colonic Tissues

**FIGURE 1:**
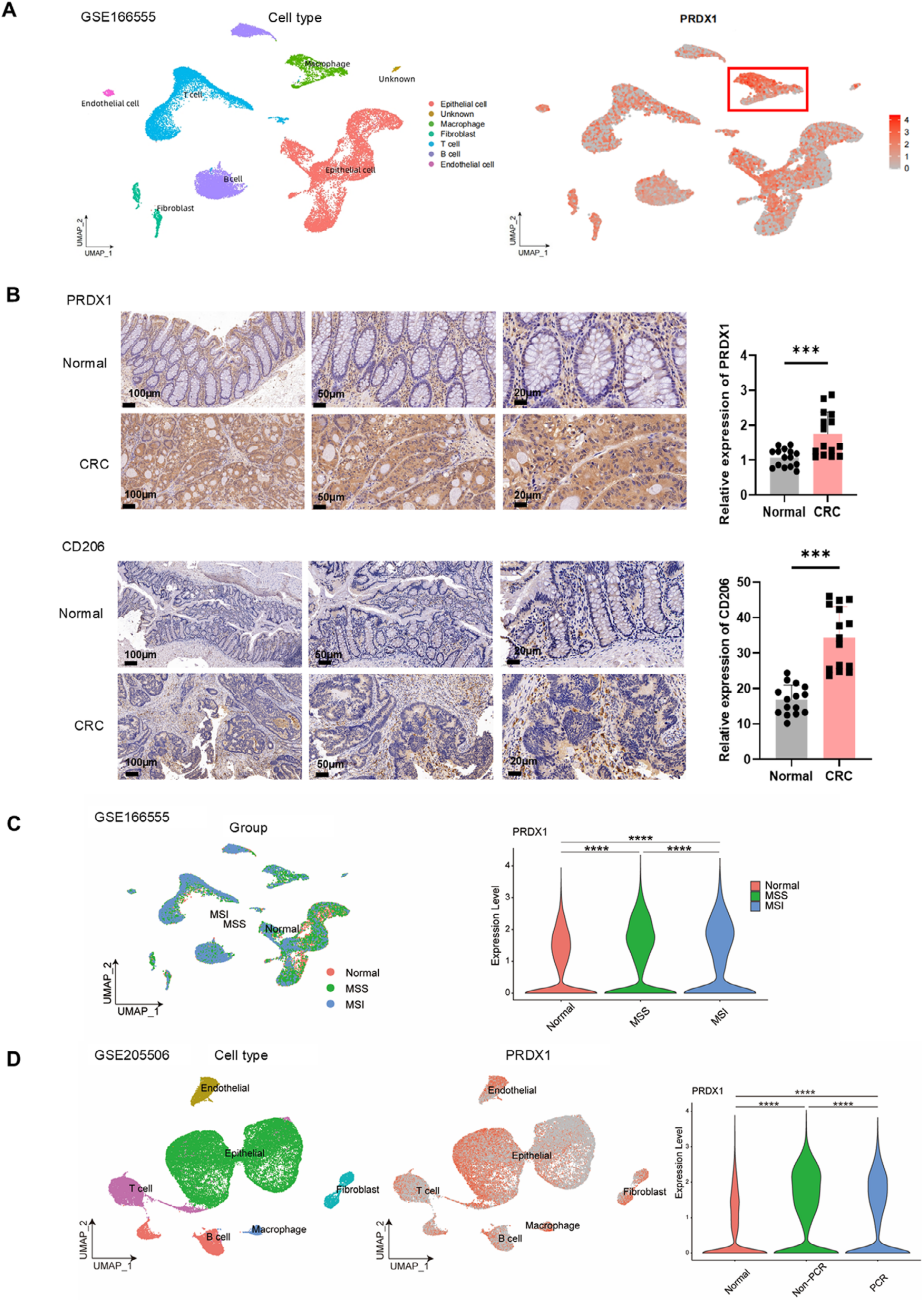
High expression of PRDX1 is associated with immune infiltration in CRC. (A) *PRDX1* expression across different cell types (epithelial, immune, and stromal cells) in CRC tissues based on the scRNA‐seq dataset GSE166555. (B) Representative IHC images showing PRDX1 and CD206 expression in paired normal colonic tissues and CRC tissues from patients. Scale bar = 100, 50, 20 µm. Data are presented as mean ± SD. ***p < 0.001, *n* = 15. (C) *PRDX1* expression levels in normal colon, MSS, and MSI‐H subtype of colorectal cancer analyzed using scRNA‐seq dataset GSE166555 (*n* = 12). (D) *PRDX1* expression levels in CRC tissues from patients showing pathological complete response (pCR, *n* = 7) or non‐pCR (*n* = 3) compared to normal colon using scRNA‐seq dataset GSE205506. ****p < 0.0001.

PRDX1 was found to be upregulated in a panel of gastrointestinal cancers including colorectal cancer (COAD, READ, and COADREAD), stomach and esophageal carcinoma (STES), stomach adenocarcinoma (STAD), and pancreatic adenocarcinoma (PAAD) using Sangerbox (http://vip.sangerbox.com/home.html) (Figure S1A). To investigate the expression of *PRDX1* across different cell types, we conducted a secondary analysis of a publicly available single‐cell RNA‐sequencing (scRNA‐seq) dataset (GSE166555), originally generated by Uhlitz et al. [15]. The dataset included tissue samples from CRC and matched normal tissues obtained from 12 previously untreated CRC patients undergoing primary surgery. Our analysis revealed that macrophages and epithelial cells exhibited significantly enriched expression of *PRDX1* compared to other cell types within the TME of colonic tissues (Figure 1A). TIMER2.0 analysis showed that overexpression of PRDX1 was negatively correlated with infiltration of CD4^+^ and CD8^+^ T cells, while positively correlated with infiltration of M2 macrophages in CRC (Figure S1B), suggesting a potential role for PRDX1 in shaping anti‐tumor immunity via macrophage polarization. To further demonstrate the clinical relevance of PRDX1 with macrophage polarization, we performed immunohistochemistry (IHC) in CRC specimens and found that both PRDX1 and CD206 were significantly upregulated in CRC tissues compared to adjacent normal tissues (Figure 1B). CD206, a specific marker of M2 polarized macrophages, was found to be predominantly located within the stromal compartment of the TME.

To explore the potential association between PRDX1 expression and sensitivity to ICI therapy in CRC patients, we further reanalyzed scRNA‐seq dataset (GSE166555) according to the MSS and MSI‐H immune subtypes. As a result, the expression of PRDX1 was higher in CRC patients with immunotherapy‐resistant MSS than in those with MSI‐H subtype (Figure 1C). To directly evaluate the relationship between PRDX1 expression and response to anti‐PD‐1 therapy, we reanalyzed another publicly available scRNA‐seq dataset (GSE205506) generated by Li et al. from 19 CRC patients receiving neoadjuvant ICIs treatment [16]. From this cohort, we focused on 10 CRC patients treated with anti‐PD‐1 monotherapy. Notably, PRDX1 expression was significantly upregulated in ICI‐resistant patients without a pathological complete response (non‐pCR; *n* = 3) compared to ICI‐sensitive patients with a pathological complete response (pCR; *n* = 7) (Figure 1D). Taken together, these results suggest that elevated PRDX1 expression is associated with resistance to immunotherapy in CRC.

**FIGURE 2:**
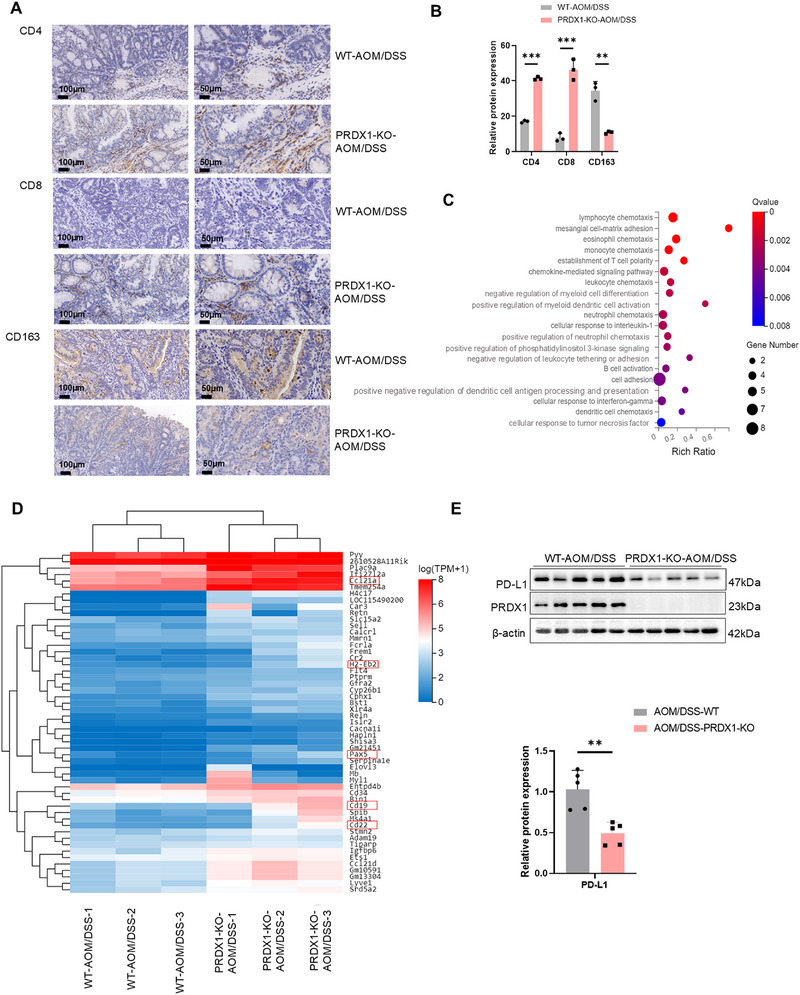
PRDX1 deficiency inhibits AOM/DSS‐induced colitis‐associated CRC and reduces M2‐TAMs infiltration in colonic tissue. (A) Representative IHC images of CD4, CD8, and CD163 in colorectal adenocarcinoma tissues from AOM/DSS‐induced WT and PRDX1 KO mice. Scale bar = 100, 50 µm. (B) Quantitative analysis of IHC staining. Data are presented as mean ± SD. ***p < 0.001. *n* = 3. (C) KEGG pathway enrichment analysis of significantly upregulated genes in CRC tissues from different groups, presented as a bubble chart. (D) Hierarchical clustering heatmap of the 53 significantly upregulated genes in CRC tissues from AOM/DSS‐induced WT and PRDX1 KO mice. (E) Western blot analysis of the expression of PD‐L1 and PRDX1 in mouse CRC tissues as indicated. **p < 0.01, *n* = 5.

To investigate whether PRDX1 promotes CRC progression by regulating tumor immune microenvironment in vivo, we treated WT and PRDX1‐KO (knockout) mice with AOM/DSS and found that the number of colitis‐associated colonic adenocarcinomas was significantly reduced in PRDX1‐KO mice compared to WT mice as described previously [11]. To assess whether PRDX1 expression influences immune cell infiltration, we performed IHC staining for T cells and the M2 macrophage marker CD163 in tumor tissues. PRDX1‐KO mice exhibited increased infiltration of CD4⁺ and CD8⁺ T cells and a concurrent reduction in M2 macrophages, as indicated by decreased CD163 expression (Figure 2A,B). We further analyzed RNA transcriptome sequencing data from colonic tissues of WT and PRDX1‐KO mice treated with AOM/DSS. There were 53 upregulated genes in colonic tissues from AOM/DSS‐treated PRDX1‐KO mice (Table S5). KEGG pathway analysis revealed enrichment in processes related to cell adhesion, immune cell chemotaxis, and B‐cell activation. Heatmap and functional annotation indicated that several significantly upregulated genes (*CCL21*, *H2‐Eb2*, *Pax5*, *CD19*, *CD22*, etc.) were associated with anti‐cancer immunity (Figure 2C,D). Gene set enrichment analysis (GSEA) further suggested that PRDX1 expression was associated with antigen processing and presentation (Figure S1C).

Western blot analysis showed that PD‐L1 protein levels were significantly decreased in colonic adenocarcinoma tissue from PRDX1‐KO mice (Figure 2E). PD‐L1, an immune checkpoint, could promote immune evasion by interacting with PD‐1 on cytotoxic T cells within tumors [17]. Thus, the downregulation of PD‐L1 suggests enhanced anti‐tumor immunity. Together, these findings indicate that PRDX1 ablation may suppress CRC development by enhancing anti‐tumor immunity through modulation of macrophage polarization and secretion of inflammatory cytokines.

### 2.2 PRDX1 Knockdown in CRC Cells Suppresses M2 Polarization of TAMs by Reducing GLUT‐1‐Mediated Lactate Secretion via Glycolysis

**FIGURE 3:**
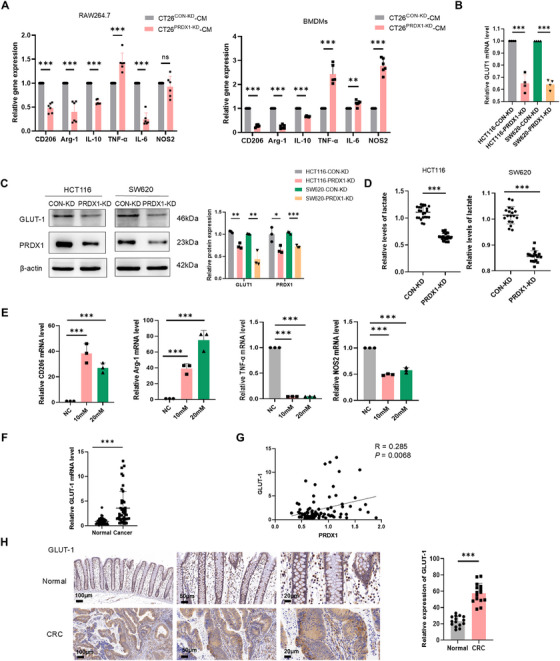
PRDX1 promotes M2 polarization of TAMs by increasing lactate levels through GLUT1‐mediated glycolysis. (A) RT‐qPCR analysis of macrophage polarization marker genes in RAW264.7 cells and BMDMs cultured with conditioned medium (CM) from CT26^PRDX1‐KD^ and CT26^CON‐KD^. Data are presented as mean ± SD. **p < 0.01, ***p < 0.001. *n* = 6. (B, C) RT‐qPCR and Western blot analysis of mRNA and protein levels of GLUT1 in HCT116 and SW620 cells infected with PRDX1‐KD or control lentivirus, with β‐actin as a loading control. Data are shown as mean ± SD. ***p < 0.001. (D) Lactate levels were determined in the SW620 and HCT116 cells infected with PRDX1‐KD or control lentivirus. (E) RT‐qPCR analysis of macrophage polarization marker genes in RAW264.7 cells stimulated with different concentrations of lactate for 24 h. Data are presented as mean ± SD. ***p < 0.001, *n* = 3. (F) RT‐qPCR analysis of the level of GLUT‐1 mRNA in 45 pairs of human CRC tissues and adjacent normal tissues. ***p < 0.001. *n* = 45. (G) Spearman correlation analysis of PRDX1 and GLUT‐1 mRNA levels in human CRC tissues (*R* = 0.285, *p* < 0.01, *n* = 45). (H) Representative IHC images of GLUT‐1 in paired human CRC tissues and adjacent normal tissues. Scale bar = 100, 50, 20 µm, ***p < 0.001, *n* = 15.

To explore the mechanism of PRDX1 on macrophage polarization, we co‐cultured RAW264.7 and bone marrow‐derived macrophages (BMDMs) with murine CT26 cells infected with PRDX1 knockdown lentivirus (CT26^PRDX1‐KD^) and vector control (CT26^CON‐KD^). The knockdown effect is shown in Figure S2A. BMDMs were identified as CD11b⁺F4/80⁺ cells by flow cytometry (Figure S2B,C). As a result, the conditioned medium (CM) from CT26^PRDX1‐KD^ cells substantially reduced the expression levels of M2 macrophage markers CD206, Arg‐1, and IL‐10, while enhancing the expression levels of M1 macrophage marker TNF‐α in both RAW264.7 and BMDMs, as determined by RT‐qPCR analysis (Figure 3A). These results suggest that PRDX1 knockdown in CRC cells impairs their ability to drive the tumor‐promoting M2 polarization of macrophages. Notably, the immune suppressive cytokine IL‐6 was also markedly suppressed in RAW264.7 cells but not in BMDMs after co‐culture with CT26^PRDX1‐KD^. Conversely, RAW264.7 cells co‐cultured with CT26^PRDX1‐OE^ cells exhibited a pronounced M2‐polarized phenotype, evidenced by increased CD206, Arg‐1, and IL‐10 mRNA levels (Figure S2D).

How do CRC cells with knockdown of PRDX1 inhibit the M2‐polarized phenotype of macrophages? RNA‐sequencing analysis revealed that PRDX1 knockdown significantly downregulated glucose transporter 1 (GLUT‐1, encoded by SLC2A1), a key regulator of glycolysis, as previously illustrated in our published study [11]. We confirmed that silencing of PRDX1 in HCT116 and SW620 cells markedly reduced GLUT‐1 expression at both mRNA and protein levels (Figure 3B,C, Figure S2E). Consistently, PRDX1 knockdown also significantly decreased the expression of key glycolysis‐associated enzymes, including hexokinase 1 (HK1), pyruvate kinase M (PKM), lactate dehydrogenase (LDHA), and the platelet isoform of phosphofructokinase 1 (PFKP) (Figure S2F,G).

Emerging evidence indicates that lactate is no longer an inert end‐product of glycolysis but an important signaling molecule in energy regulation, immune tolerance, and cancer growth [18]. Previous studies have demonstrated that tumor‐derived lactate induced M2 macrophage polarization in both BMDMs and THP‐1 cells [19, 20]. To investigate whether PRDX1 enhances lactate secretion by upregulating GLUT‐1‐mediated glycolysis, we measured lactate levels in SW620^PRDX1‐KD^, HCT116^PRDX1‐KD^, and their corresponding control cells using spectrophotometry. As expected, PRDX1 knockdown led to a significant reduction in lactate levels compared to control cells (Figure 3D). Importantly, overexpression of GLUT1 in SW620^PRDX1‐KD^ cells rescued lactate secretion (Figure S2H), suggesting that PRDX1 regulates lactate secretion primarily through GLUT1‐mediated glycolysis. Consistent results were observed in murine CRC cells, with CT26^PRDX1‐KD^ showing decreased lactate levels and CT26^PRDX1‐OE^ showing increased lactate secretion (Figure S2H).

To assess the functional effect of lactate on macrophage polarization, we treated macrophage with different concentrations of lactate. Notably, lactate treatment markedly upregulated the expression of M2 markers (*CD206* and *Arg‐1*), while significantly downregulated M1 markers (*TNF‐α* and *NOS2*) (Figure 3E), highlighting the pivotal role of lactate in promoting M2 polarization. Intriguingly, we also observed that lactate increased PRDX1 mRNA levels in macrophages (Figure S2I), suggesting a potential feedback mechanism that could influence macrophage polarization. However, the molecular mechanism remains to be fully elucidated.

To further validate the association between PRDX1 and GLUT‐1 expression, we performed RT‐qPCR analysis in 45 pairs of clinical CRC specimens. The results revealed significantly elevated GLUT‐1 mRNA expression in CRC tissues compared to adjacent normal colonic tissues (Figure 3F). Furthermore, a significant positive correlation was observed between PRDX1 and GLUT‐1 mRNA expression levels (*R* = 0.285, *p* < 0.01) (Figure 3G). IHC staining further confirmed that GLUT‐1 protein was highly expressed in CRC tissues, with predominant localization to the plasma membrane (Figure 3H). These results demonstrate that PRDX1‐overexpressing CRC cells promote M2 macrophage polarization by enhancing lactate secretion through GLUT‐1‐mediated glycolysis.

### 2.3 PRDX1 Enhances GLUT‐1‐Mediated Tumor Glycolysis by Inhibiting HIF1α Ubiquitination and Degradation Through Binding to Cullin‐2

**FIGURE 4:**
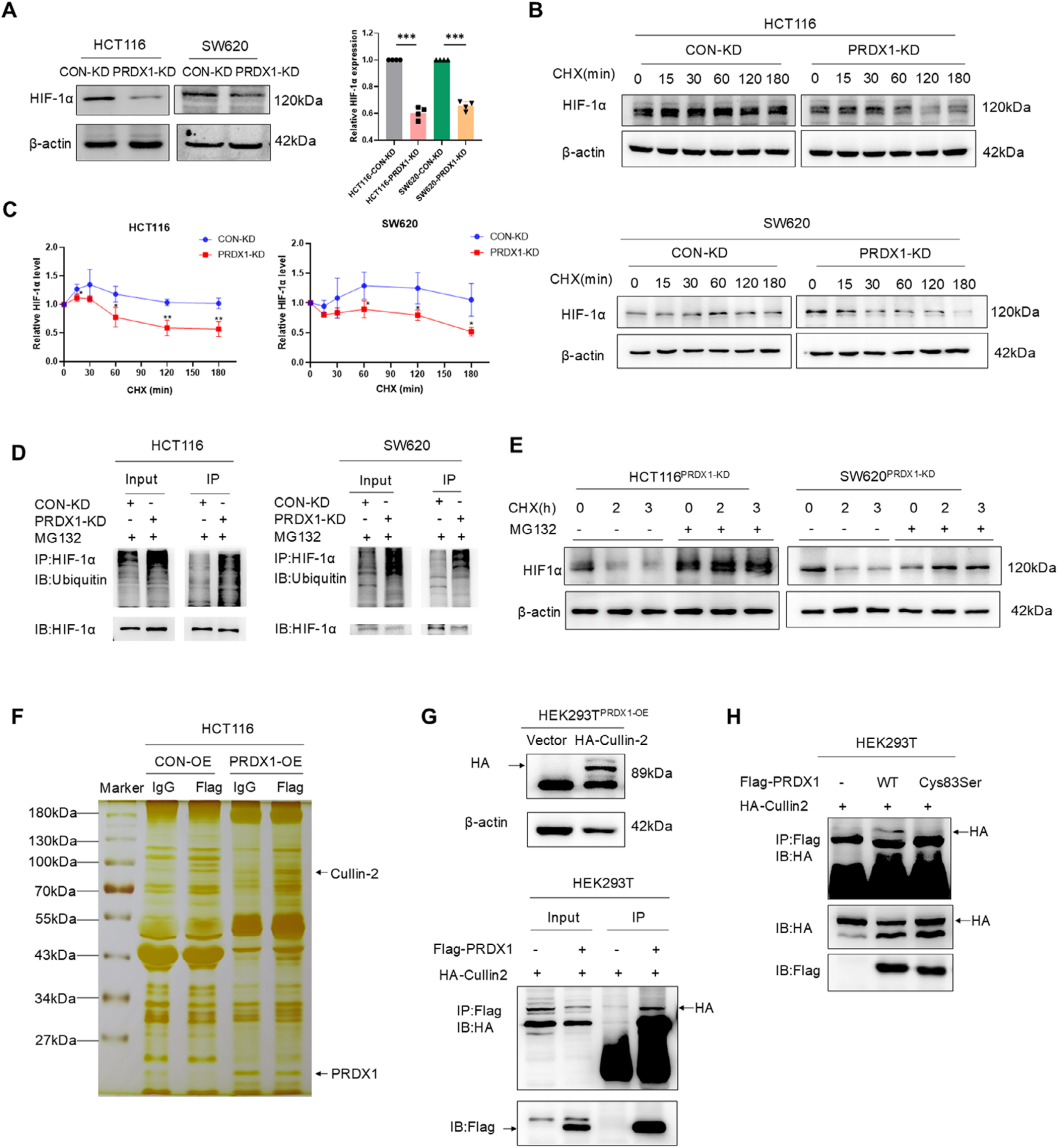
PRDX1 enhances glycolysis through inhibiting HIF‐1α ubiquitination and degradation by binding to Cullin‐2. (A) Western blot analysis of HIF‐1α protein levels in HCT116^PRDX1‐KD^ and SW620^PRDX1‐KD^ cells compared to their respective controls. β‐Actin was used as a loading control. Data are presented as mean ± SD. ***p < 0.001, *n* = 3. (B, C) CHX chase assay was performed to evaluate the degradation of HIF‐1α in HCT116 and SW620 cells with PRDX1 knockdown compared to negative controls. The degradation curves of HIF‐1α are shown. (D) Immunoprecipitation assay was used to detect the ubiquitination of HIF‐1α in CRC cells with PRDX1 knockdown compared to controls. (E) Western blot analysis of HIF‐1α in HCT116^PRDX1‐KD^ and SW620^PRDX1‐KD^ cells treated with 100 µg/mL CHX for indicated durations, with or without the pretreatment of MG132 (20 µM) for 6 h. (F) Silver staining of proteins co‐immunoprecipitated with PRDX1, showing potential interacting proteins. (G) Western blot analysis of HA expression in HEK293^PRDX1‐OE^ cells transfected with HA‐Cullin‐2 plasmid and empty vector, with β‐actin as a loading control. Co‐IP analysis of the interaction between Flag‐PRDX1 and HA‐Cullin‐2 in HEK293T or HEK293^PRDX1‐OE^ cells transfected with HA‐Cullin‐2 as indicated. (H) Co‐IP analysis of the interaction between Flag‐PRDX1 and HA‐Cullin‐2 in HEK293^PRDX1‐WT^ or HEK293^PRDX1‐Cys83Ser^ cells transfected with HA‐Cullin‐2 as indicated.

HIF‐1α has been shown to be the key transcriptional factor orchestrating the expression of the target gene GLUT‐1 [21]. To investigate whether PRDX1 regulates glycolysis through the HIF‐1α/GLUT‐1 signaling pathway, we first examined HIF‐1α protein expression. Western blot analysis showed that HIF‐1α levels were significantly decreased in HCT116^PRDX1‐KD^ and SW620^PRDX1‐KD^ cells compared to their respective control cells (Figure 4A). Specifically, overexpression of HIF‐1α in SW620^PRDX1‐KD^ cells rescued the expression of GLUT1 (Figure S3A). Consistently, PRDX1 knockdown in murine CT26 cells reduced both HIF‐1α and GLUT‐1 protein levels, whereas PRDX1 overexpression increased their expression (Figure S3B). These results indicate that PRDX1 regulates glycolysis‐mediated lactate production in CRC cells through HIF‐1α/GLUT‐1 signaling pathway.

Besides its antioxidant function, PRDX1 exhibits molecular chaperone activity that modulates protein stability [10]. Cycloheximide (CHX)‐chase assays revealed that PRDX1 knockdown accelerated HIF‐1α degradation in SW620 and HCT116 cells (Figure 4B,C), whereas PRDX1 overexpression in SW480 cells attenuated HIF‐1α turnover (Figure S3C). We next assessed whether PRDX1 affects HIF‐1α ubiquitination. Expectedly, silencing of PRDX1 markedly reinforced the ubiquitin conjugation to HIF‐1α, thereby enhancing the ubiquitin‐mediated degradation of HIF‐1α (Figure 4D). Generally, the Cullin‐2‐RING E3 ligase complex plays a critical role in oxygen sensing by targeting HIF‐1α for ubiquitination and degradation [22]. As shown in Figure 4E, MG132 significantly suppressed the ubiquitination‐mediated degradation of HIF‐1α. Silver staining of proteins in polyacrylamide gels and silver staining and immunoprecipitation‐mass spectrometry (IP‐MS) results revealed that there was a panel of proteins that could interact with PRDX1 including Cullin‐2 (Figure 4F). The IP‐MS results were listed in our previous paper [11]. To investigate whether PRDX1 regulates HIF‐1α ubiquitination and degradation by binding to Cullin‐2, we performed co‐immunoprecipitation (Co‐IP) analysis and found that Flag‐PRDX1 physically interacts with HA‐Cullin‐2 to form a degradation complex (Figure 4G). Notably, this interaction was significantly abolished by mutation of Cys83 in PRDX1 (Figure 4H). These results demonstrate that PRDX1 functions as a molecular chaperone that binds to Cullin‐2, thereby regulating HIF‐1α/GLUT1‐mediated glycolysis.

### 2.4 PRDX1 Deficiency in Macrophages Promotes Polarization to M1‐Like Phenotypes

Studies have shown that PRDX1 is the most highly expressed antioxidant enzyme in macrophages, and its deficiency leads to excessive oxidative stress and disrupted autophagic flux maintenance in these cells [23]. We have previously demonstrated that CRC cells overexpressing PRDX1 promote M2 macrophage polarization by increasing lactate secretion via HIF‐1α/GLUT‐1 signaling pathways. To investigate whether PRDX1 silencing in macrophages reciprocally influences CRC progression, we further analyzed the scRNA‐seq dataset (GSE166555) and observed higher PRDX1 expression in TAMs from CRC tissues compared to those in adjacent normal colonic tissues (Figure S3D).

**FIGURE 5:**
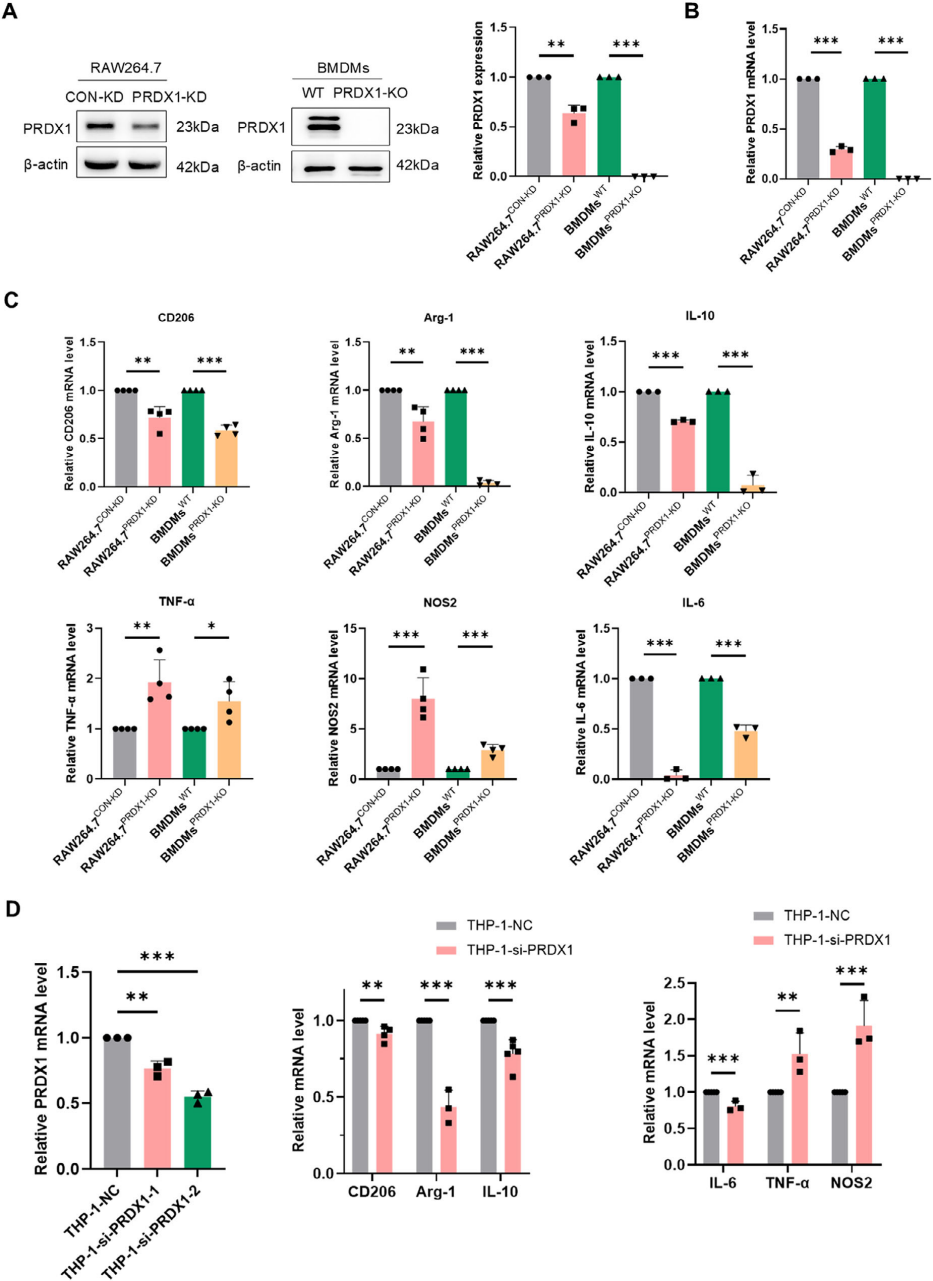
PRDX1 deficiency in macrophages promotes polarization to M1‐like phenotypes. (A, B) Western blot and RT‐qPCR analysis of protein and mRNA levels of PRDX1 in RAW264.7 and BMDMs, respectively. (C) RT‐qPCR analysis of mRNA levels of M1 and M2 macrophage polarization markers in PRDX‐deficient RAW264.7 cells and BMDMs compared to controls. (D) RT‐qPCR analysis of mRNA levels of macrophage polarization markers in THP‐1 cells transfected with si‐PRDX1 or negative control siRNA (si‐NC). Data are presented as mean ± SD.*p < 0.05, **p < 0.01, ***p < 0.001, *n* = 3.

To determine the potential impact of PRDX1 on macrophage polarization in vitro, we knocked down PRDX1 in murine macrophages RAW264.7 and further isolated BMDMs from PRDX1‐KO and WT mice (Figure 5A,B). Polarization phenotypes were assessed by measuring the expression of a panel of marker genes in both RAW264.7 and BMDMs by RT‐qPCR analysis. As shown in Figure 5C, the expression levels of CD206, arginase 1 (Arg‐1), and IL‐10, the classical M2 markers, were markedly reduced compared to negative controls, whereas the expression levels of NOS2 and TNF‐α, the classical M1 markers, were significantly increased in BMDMs^PRDX1‐KO^ and RAW264.7^PRDX1‐KD^ cells. It has been reported that IL‐6 could be an immunosuppressive inflammatory cytokine involved in both M1 and M2 polarization of macrophages [24]. Particularly, knockdown of PRDX1 in macrophages led to a significant reduction in the level of IL‐6. Flow cytometry analysis further confirmed that there was a significant reduction in the proportion of F4/80^+^CD206^+^ M2 macrophages among PRDX1‐deficient BMDMs (Figure S3E). These results indicate that PRDX1 depletion in macrophages promotes their polarization toward M1‐like phenotypes, highlighting a critical role for PRDX1 in regulating macrophage function and anti‐tumor immunity.

To explore whether PRDX1 plays a similar role in human macrophages, we knocked down PRDX1 in PMA‐induced THP‐1 cells using siRNAs. As expected, expression levels of M1 markers (TNF‐α and NOS2) were upregulated while M2 markers (Arg‐1 and IL‐10) were significantly decreased in PRDX1‐silenced THP‐1 cells (Figure 5D). These results substantiate an important role of PRDX1 in promoting M2 polarization of TAMs.

### 2.5 PRDX1 Deficiency Enhances Macrophage Phagocytosis and Suppresses Cancer Cell Proliferation and Migration

**FIGURE 6:**
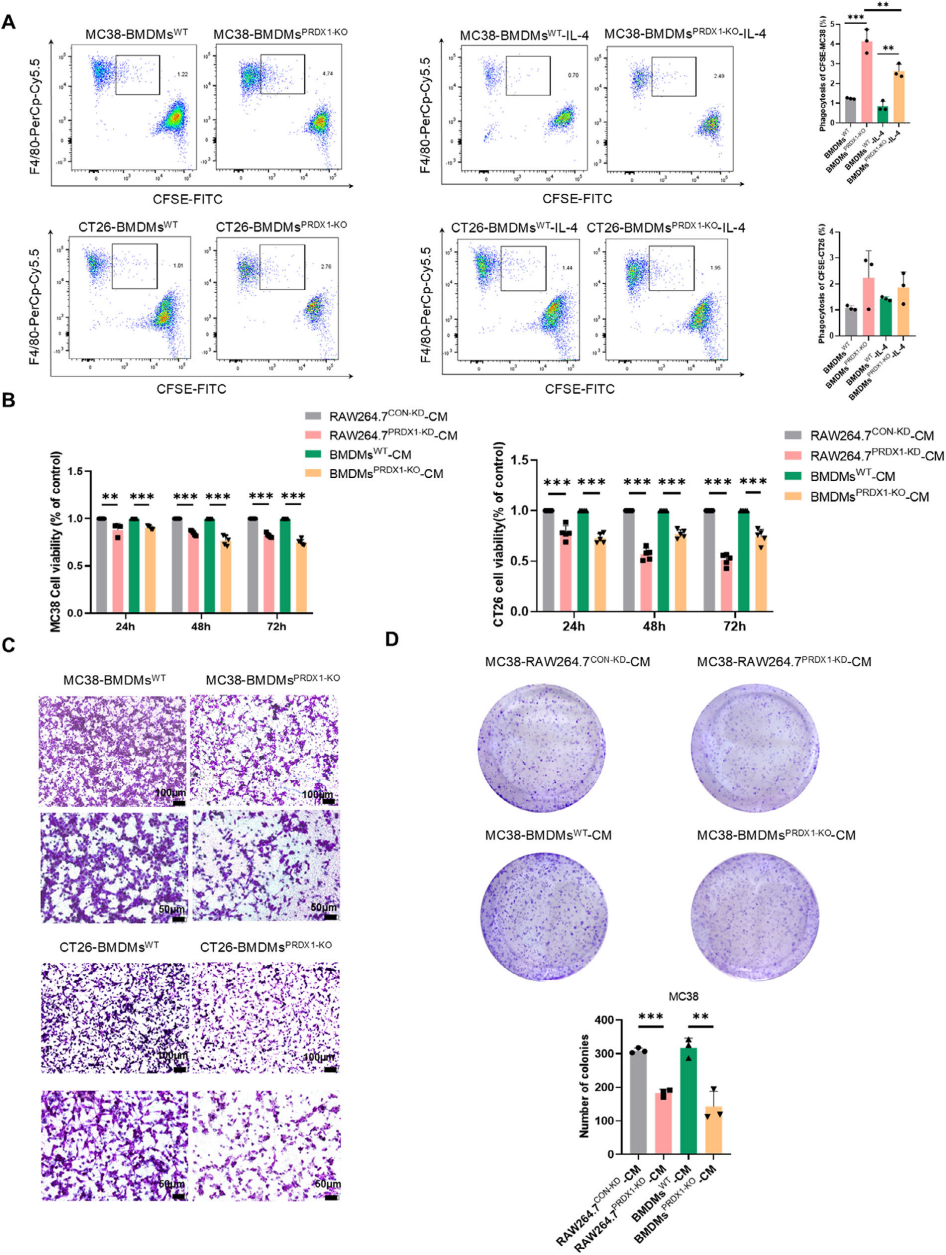
PRDX1 deficiency enhances macrophage phagocytosis and inhibits cancer cell proliferation and migration. (A) Flow cytometry analysis of the phagocytosis capacity for CFSE‐labeled MC38 and CT26 cells by BMDMs^WT^ and BMDMs^PRDX1‐KO^ treated with or without IL‐4. (B) MTT assay was performed to determine the cell viability of MC38 and CT26 cells treated with CM from RAW264.7^PRDX1‐KD^ or BMDMs^PRDX1‐KO^ and their respective controls for different time periods. (C, D) Transwell and colony formation assays were performed to determine the cell migration and proliferation of CRC cells treated with CM as indicated. Data are presented as mean ± SD, **p < 0.01, ***p < 0.001, *n* = 3.

Upon exposure to tumor antigens, macrophages phagocytose and present these antigens to T cells, enhancing anti‐tumor immune responses through co‐stimulatory signaling [25]. After phagocytosis of tumor cells, TAMs shifted toward an M2 polarized phenotype and exhibited pro‐tumor effects. We next assessed whether PRDX1 could affect the macrophages phagocytosis of tumor cells by flow cytometry. CFSE‐labeled MC38 or CT26 cells were co‐cultured with BMDMs^WT^ and BMDMs^PRDX1‐KO^ with or without the induction of IL‐4. The results indicated that BMDMs^PRDX1‐KO^ exhibited increased phagocytosis of both MC38 and CT26 cells compared to BMDMs^WT^, which was attenuated by treatment with the M2 macrophage‐inducing cytokine IL‐4 (Figure 6A).

In order to explore the effects of PRDX1‐KO macrophages on the proliferation, migration, and colony‐formation capacity of CRC cells, murine MC38 and CT26 cells were treated with CM from PRDX‐KO macrophages and controls. As shown in Figure 6B, there was a marked reduction in cell viability in a time‐dependent manner when incubated with CM from both BMDMs^PRDX1‐KO^ and RAW264.7^PRDX1‐KD^ compared to CM from control cells. Consistently, transwell migration assays and colony formation assays revealed that CM from BMDMs^PRDX1‐KO^ and RAW264.7^PRDX1‐KD^ markedly inhibited the migration and colony formation capacities of MC38 or CT26 cells (Figure 6C,D). Taken together, these results indicate that PRDX1‐KO macrophages exhibited enhanced phagocytosis and consequently suppressed tumor cell proliferation and migration in a co‐culture system.

### 2.6 PRDX1 Ablation in Macrophages Restricts Tumor Growth by Controlling the Secretion of Cytokines Through JAK/STAT1/NF‐κB Pathways

**FIGURE 7:**
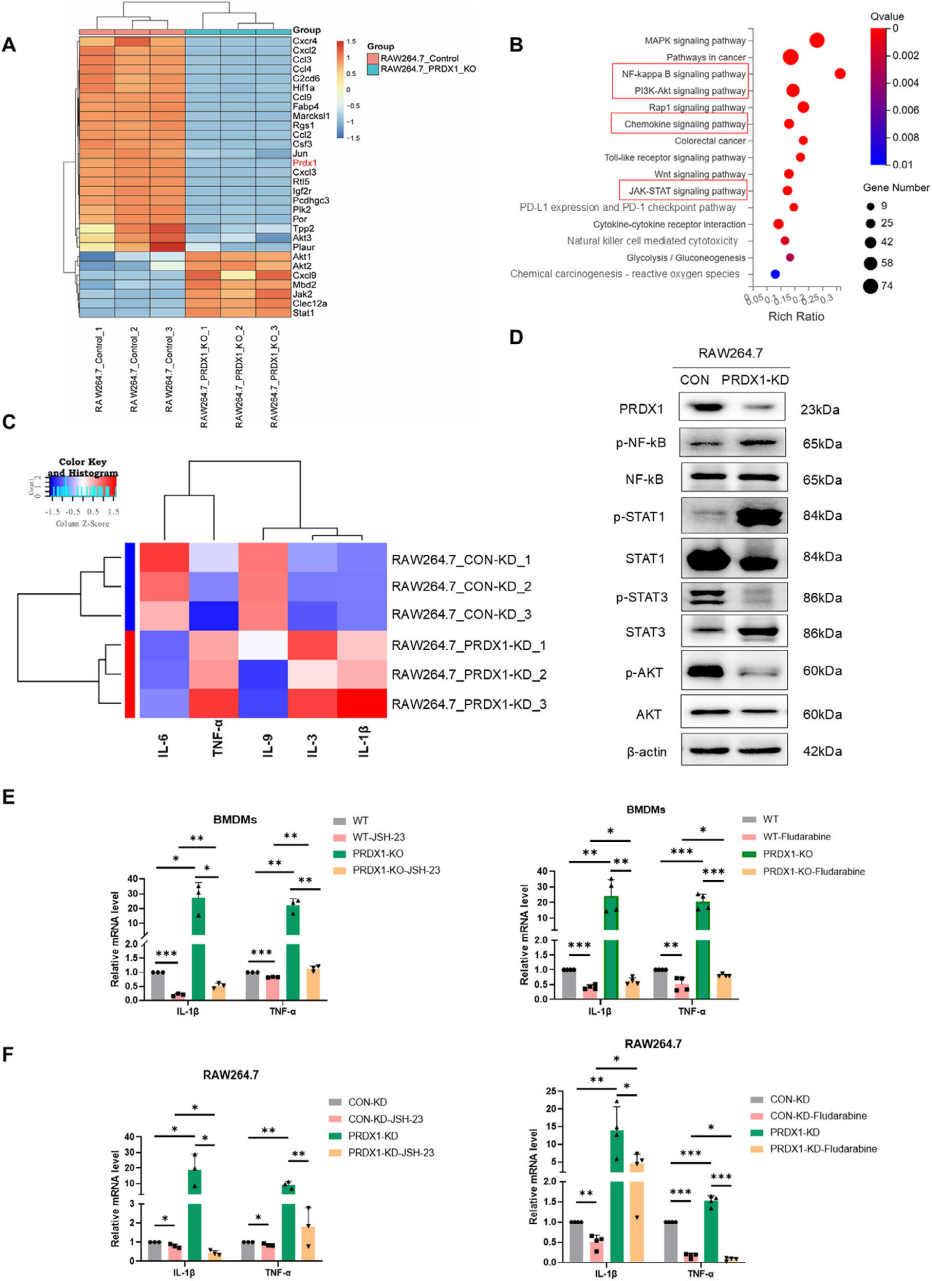
PRDX1 deficiency in macrophages promotes M1 polarization and enhances TNF‐α/IL‐1β secretion through the JAK/STAT1/NF‐κB pathway. (A) Hierarchical clustering analysis of differentially expressed mRNAs between RAW264.7^CON‐KD^ and RAW264.7^PRDX1‐KD^ cells. (B) KEGG pathway enrichment analysis of differentially expressed genes between RAW264.7^CON‐KD^ and RAW264.7^PRDX1‐KD^ cells. (C) Heatmap showing differential expression of cytokines in RAW264.7^CON‐KD^ versus RAW264.7^PRDX1‐KD^ cells. (D) Western blot analysis of protein expression in RAW264.7^CON‐KD^ and RAW264.7^PRDX1‐KD^ cells. (E, F) RT‐qPCR analysis of IL‐1β and TNF‐α mRNA levels in BMDMs^WT^ and BMDMs^PRDX1‐KO^ or RAW264.7^CON‐KD^ and RAW264.7^PRDX1‐KD^ cells, treated with or without NF‐κB inhibitor JSH‐23 (60 µM) or STAT1 inhibitor fludarabine (5 µM) for 24 h. Data are shown as mean ± SD. *p < 0.05, **p < 0.01, ***p < 0.001, *n* = 4.

Macrophages exert cytotoxic effects on tumor cells by secreting inflammatory cytokines. To explore the signaling pathways involved in the secretion of inflammatory cytokines, we performed RNA sequencing in RAW264.7^CON‐KD^ and RAW264.7^PRDX1‐KD^ cells. There were totally 348 upregulated genes and 738 downregulated genes (|log_2_ (RAW264.7‐PRDX1‐KD/Control)| ≥ 1, adjusted *p* < 0.05) between the two groups (Table S6). KEGG pathway analysis indicated significant enrichment in JAK‐STAT1, NF‐κB, PI3K‐Akt, and chemokine signaling pathways (Figure 7A,B). Interestingly, subsequent GSEA revealed that PD‐L1 expression and PD‐1 checkpoint pathway were associated with PRDX1 silencing in RAW264.7 cells, implying that dysregulation of PRDX1 in macrophages possibly affects the efficacy of immunotherapy (Figure S4A,B).

To delineate the exact cytokines secreted by RAW264.7^PRDX1‐KD^ cells that reciprocally suppress tumor growth, we conducted a mouse cytokine antibody array and semi‐quantitatively measured 20 inflammatory cytokines in the CM of macrophages. As a result, the levels of TNF‐α, IL‐1β, and IL‐3 were significantly elevated, whereas IL‐6 and IL‐9 were markedly reduced in CM of RAW264.7^PRDX1‐KD^ cells (Figure 7C), suggesting that inflammatory signaling pathways possibly orchestrate the polarization phenotype and cytokine secretion of macrophages (Table S7). Consistently, cytokines such as IL‐1β, IFN‐γ, and IL‐12 were also significantly upregulated in BMDMs^PRDX1‐KO^ compared to BMDMs^WT^ (Figure S4C, Table S8). Western blot analysis indicated that activation of JAK‐STAT1/NF‐κB signaling and inhibition of PI3K‐Akt signaling played an important role in restraining M2 polarization of macrophages. The activation of JAK‐STAT1/NF‐κB signaling was responsible for increased secretion of the inflammatory cytokines IL‐1β and TNF‐α in CM from RAW264.7^PRDX1‐KD^ and skewed macrophages toward M1 polarization (Figure 7D). To confirm this, we performed RT‐qPCR analysis of the expression of IL‐1β and TNFα mRNA in BMDMs^WT^ and BMDMs^PRDX1‐KO^ with or without the treatment of JSH‐23, an NF‐κB inhibitor that blocks its transcriptional activity. As expected, KO of PRDX1 significantly upregulated the mRNA levels of IL‐1β and TNFα, which were strongly impaired by the treatment of JSH‐23. The STAT1 inhibitor fludarabine also resulted in a significant reduction of IL‐1β and TNFα mRNA levels in BMDMs^PRDX1‐KO^ (Figure 7E). Consistently, similar results were found in RAW264.7^PRDX1‐KD^ versus RAW264.7^CON‐KD^ cells treated with JSH‐23 or fludarabine (Figure 7F). These results revealed that PRDX1 ablation promotes M1 macrophage polarization and enhances secretion of IL‐1β and TNF‐α through activation of JAK‐STAT1 and NF‐κB signaling, thereby suppressing CRC progression.

### 2.7 Depletion of PRDX1 in Macrophages Inhibits CRC Growth and Sensitizes to PD‐1 Therapy by Potentiating CD8^+^ T Cells Function

To investigate whether PRDX1 deficiency in macrophages inhibits CRC growth by enhancing T‐cell‐mediated cytotoxicity, we mixed MC38 cells with BMDMs^PRDX1‐KO^ cells or BMDMs^WT^ cells (3:1 ratio) and injected subcutaneously into C57BL/6J mice pre‐cleared of macrophages by clodronate liposomes. The clearance efficiency of macrophages is shown in Figure S4D. The results showed that co‐injection of BMDMs^PRDX1‐KO^ cells significantly suppressed MC38 cell syngeneic tumor growth (Figure S5A).

Since T lymphocytes function as the predominant tumoricidal effector cells, we hypothesized that PRDX1 ablation in macrophages enhanced anti‐tumor immunity by inducing an M1 polarization phenotype. Therefore, we simultaneously stained CD4^+^ and CD8^+^ T cells and CD206^+^ M2 TAMs in the syngeneic tumor tissues using multiplex Immunofluorescence (mIF). Notably, the number of CD206^+^ M2 TAMs was significantly reduced, whereas the number of tumor‐infiltrating CD4^+^ and CD8^+^ T cells was markedly increased in syngeneic tumor tissues generated by co‐injection of BMDMs^PRDX1‐KO^ cells (Figure S5B,C). Supportively, there was a marked increase in M1‐polarized macrophages, as indicated by prominent elevation in the percentage of CD11b^+^F4/80^+^CD86^+^ cells and a reduction in the percentage of CD11b^+^F4/80^+^CD206^+^ cells in the BMDMs^PRDX1‐KO^ group compared to the BMDMs^WT^ group by flow cytometry (Figure S5D). To further determine the polarization of BMDMs^PRDX1‐KO^ cells on tumoricidal functions of T cells, we subsequently examined the expression of cytokines IFN‐γ, TNF‐α, granzyme B (GzmB), and PD‐1 in infiltrating CD8^+^ T cells. The expressions of IFN‐γ, TNF‐α, and GzmB were significantly increased, while PD‐1 was dramatically decreased in CD8^+^ T cells from tumor tissues of BMDMs^PRDX1‐KO^ group compared to the BMDMs^WT^ group (Figure S5E).

**FIGURE 8:**
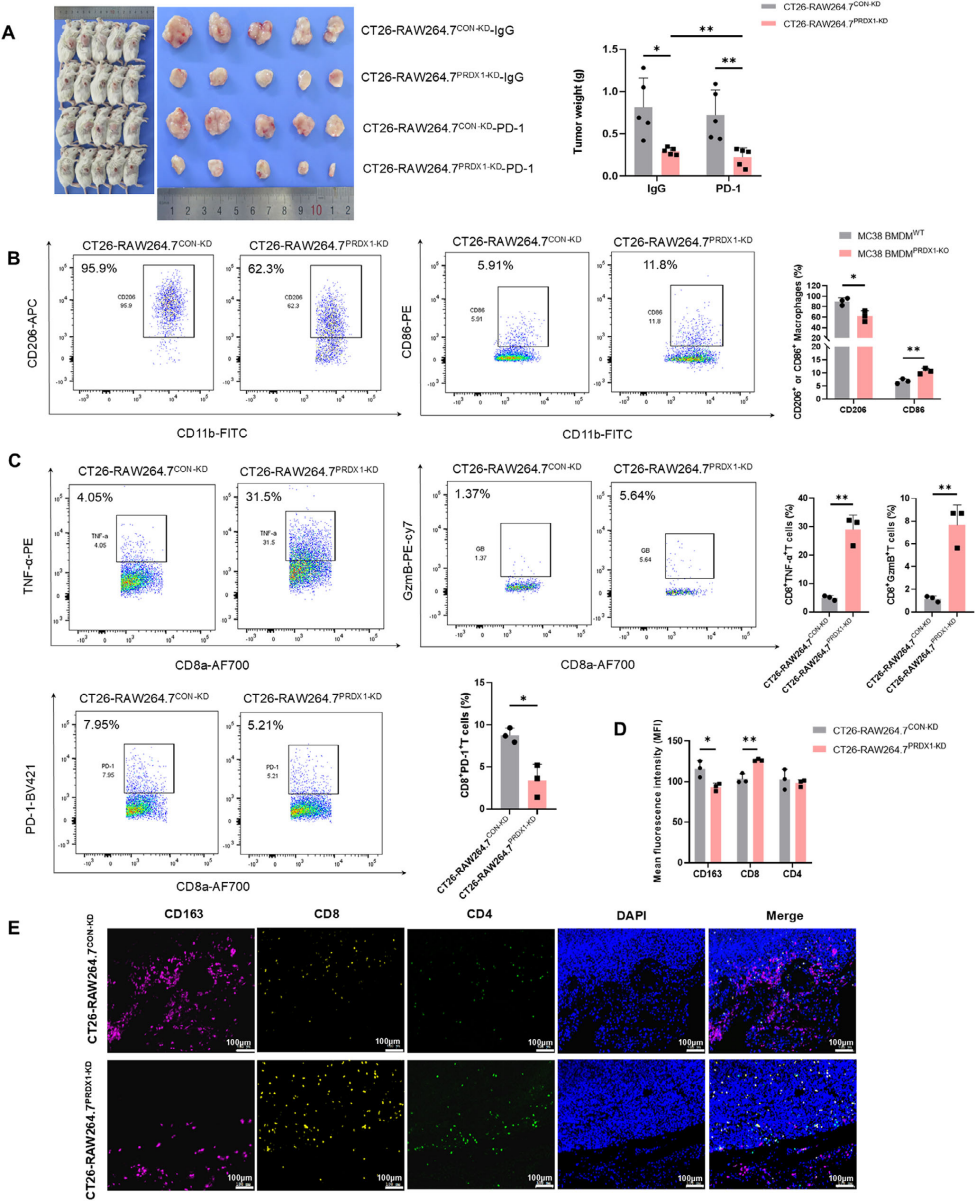
PRDX1 Deficiency inhibits colorectal cancer tumorigenesis and potentiates PD‐L1 blockade by suppressing M2 macrophage polarization in vivo. (A) Representative images of syngeneic tumors in Balb/c mice inoculated with CT26 cells and RAW264.7^CON‐KD^ and RAW264.7^PRDX1‐KD^ (3:1 ratio), followed by treatment with anti‐PD‐1 or control IgG antibodies. The tumor weight was measured and data are presented as mean ± SD, **p < 0.01, *n* = 5. (B, C) Flow cytometry analysis of the expression of CD206‐APC and CD86‐PE in CD11b^+^/F4/80^+^ macrophages as well as the expression of TNF‐α‐PE, GzmB‐PE‐Cy7, and PD‐1‐BV421 in CD8^+^ T cells. Statistical analysis was performed between the groups. *p < 0.05, **p < 0.01, ***p < 0.001, *n* = 3. (D, E) mIF analysis of CD163 (purple), CD8 (yellow), and CD4 (green) in tumor tissues. Scale bar = 100 µm.

To further investigate whether PRDX1 loss in macrophages enhances the efficacy of anti‐PD‐1 immunotherapy in vivo, we inoculated CT26 cells into Balb/c mice together with RAW264.7^CON‐KD^ and RAW264.7^PRDX1‐KD^ cells, respectively. After 1 week, both of the groups were injected intraperitoneally with anti‐PD‐1 antibody or anti‐IgG control antibody. As a result, RAW264.7^PRDX1‐KD^ dramatically inhibited syngeneic tumor growth and increased tumor sensitivity to anti‐PD‐1 antibody therapy compared to the RAW264.7^CON‐KD^ group (Figure 8A). Consistently, flow cytometry analysis indicated that macrophages were skewed toward the M1 phenotype, as indicated by increased percentages of CD11b^+^F4/80^+^CD86^+^ cells and reduced percentages of CD11b^+^F4/80^+^CD206^+^ cells in syngeneic tumor tissues of the RAW264.7^PRDX1‐KD^ group compared to the RAW264.7^CON‐KD^ control group (Figure 8B). Similarly, knockdown of PRDX1 in RAW264.7 augmented the cytotoxicity of tumor‐infiltrating CD8^+^ T cells as shown by enhanced expression of TNF‐α and GzmB, concurrent with reduced expression of PD‐1 (Figure 8C). Although CT26 cells are mismatch repair‐proficient (pMMR)/MSS and typically resistant to anti‐PD‐1 monotherapy, PRDX1 knockdown in macrophages sensitized them to anti‐PD‐1 treatment. Multiplex immunofluorescence confirmed that RAW264.7^PRDX1‐KD^ cells enhanced anti‐tumor immunity by increasing the infiltration and improving the function of CD8^+^ T cells (Figure 8D,E). These results demonstrate that reprogramming of TAMs to M1 phenotype by deletion of PRDX1 effectively suppresses CRC growth and improves the efficacy of ICIs by enhancing CD8⁺ T‐cell‐mediated anti‐tumor immunity.

## 3 Discussion

Although PD‐1/PD‐L1 immune checkpoint blockade has displayed clinical efficacy, an increasing number of patients show poor response to immunotherapy and develop resistance. TAMs, as the most abundant tumor‐infiltrating immune cells, have been proposed to be closely associated with tumor metastasis and immune escape [26]. Rather than depleting TAMs entirely, current therapeutic strategies focus on metabolic changes and functional plasticity, converting M2‐like TAMs into anti‐tumor M1‐like phenotype [27]. Therefore, reprogramming macrophage polarization represents a promising strategy for overcoming immunosuppression and enhancing anti‐tumor immunity.

PRDX1, a key member of the peroxidase family, exhibits potent antioxidant activity through scavenging reactive oxygen species (ROS). Notably, PRDX1 also functions as a molecular chaperone, playing a context‐dependent dual role in either promoting or suppressing tumor progression [10]. It has recently been reported that celastrol suppresses CRC via covalent targeting of ROS‐manipulating PRDX1 [28], indicating the potential of PRDX1 as a molecular target for cancer therapy. Single‐cell RNA‐sequencing datasets from CRC patients (GSE205506) provide a potential link between PRDX1 overexpression and resistance to anti‐PD‐1 immunotherapy. In this study, we unveil a novel role of PRDX1 in promoting CRC progression and immunotherapy resistance through crosstalk between CRC cells and TAMs. Notably, knockdown of PRDX1 in CRC cells reduces lactate secretion by suppressing HIF‐1α/GLUT‐1‐mediated glycolysis, thereby inhibiting the M2 polarization of TAMs. This effect occurs through disruption of the molecular chaperone function of PRDX1, in which the Cys83 residue is critical for binding to Cullin‐2 and stabilizing HIF‐1α. These results indicate that targeting PRDX1 in cancer cells may serve as a potential therapeutic strategy to suppress M2 macrophage polarization.

Research on the Warburg effect, a hallmark metabolic reprogramming in tumors, has identified multiple therapeutic agents targeting glucose metabolism including GLUT‐1 inhibitors with potential anticancer activity [29].  GLUT‐1, a key protein in glycolysis, was found to be downregulated in HCT116^PRDX1‐KD^ cells, suggesting that PRDX1 may regulate the expression of GLUT‐1 in cancer cells. Accumulating evidence indicates that HIF‐1α is strongly associated with the expression of target gene GLUT‐1, thereby enhancing glycolytic capacity [21, 30]. Neddylation is a post‐translational modification involving the conjugation of the ubiquitin‐like molecule NEDD8 to specific target proteins, regulating their stability or degradation. Among these targets, Cullins, particularly Cullin‐2 (CUL2), play a critical role in modulating HIF‐1α stability [31]. CUL2, in complex with von Hippel–Lindau protein (pVHL), forms an E3 ubiquitin ligase that ubiquitinates HIF‐1α, targeting it for proteasomal degradation and thereby suppressing downstream signaling pathways [31, 32]. In this study, our findings revealed that PRDX1 can bind to CUL2 and prevent the formation of CUL2‐pVHL E3 ligase complex to ubiquitinate HIF‐1α, resulting in increased HIF‐1α stability. Therefore, PRDX1 could regulate HIF‐1α/GLUT‐1‐mediated glycolysis and lactate secretion by binding to CUL2 as a molecular chaperone.

The molecular chaperone activity of PRDX1 has attracted significant attention for its role in cancer growth and drug resistance [33]. It has been proposed that PRDX1 can be oligomerized under oxidative stress to function as a molecular chaperone that could interact with c‐Myc, p53, or nuclear transcription factors such as nuclear factor kappa B (NF‐κB) [34]. Our results indicated that PRDX1 could bind to CUL2, which was abolished by mutation of Cys83. These results were consistent with the established view that Cys83 at the putative dimer–dimer interface might influence the oligomeric structure and chaperone activity of decameric PRDX1 [35]. The chaperone activity of PRDX1 increased concurrently with the loss of its peroxidase activity [36]. Structural analyses indicate that PRDX1 can assemble into either homodimers or doughnut‐shaped homodecamers. Further studies are warranted to investigate whether hyperoxidation of PRDX1 at Cys52 and Cys173 exerts effects on the interaction between PRDX1 and CUL2.

TAMs account for 30%–50% of the immune cells in most of tumor tissues and play an important role in both innate and acquired immunity by remodeling the tumor immune microenvironment [27]. Consequently, TAMs have emerged as a key therapeutic target in anti‐tumor immunotherapy, particularly through strategies aimed at reprogramming M2‐like TAMs to an M1‐like phenotype. M1 macrophages exert tumoricidal effects through mechanisms such as phagocytosis and pro‐inflammatory cytokine secretion, whereas M2 macrophages, which are often enriched in tumors, promote tumor progression and immune evasion. There are multiple signaling pathways and transcription factors that rigorously orchestrate the process of macrophage polarization. It has been reported that PI3K‐AKT, JAK‐STAT6, and PPARγ promote M2 polarization of macrophages, while TLR4/NF‐κB and JAK‐STAT1 signaling are involved in the M1 polarization [37]. Notably, it has been proposed that lactate from glycolysis could skew TAMs toward an immunosuppressive M2 polarization in cancer [38].

In this study, we found that knockdown of PRDX1 in CRC cells led to a significant suppression of glycolysis through the HIF‐1α/GLUT‐1 pathway, thereby reducing lactate production and subsequently inhibiting the polarization of macrophages toward the M2 phenotype. Conversely, overexpression of PRDX1 produced the opposite effect, enhancing glycolytic activity and promoting M2 macrophage polarization. Lactate, not only a metabolic by‐product of glycolysis, but also a signaling molecule, gets involved in the M2‐like polarization of TAMs and immunosuppression [38, 39]. Lactate has been shown to function as a signaling molecule, driving M2 polarization of TAMs through the transcription factor HIF‐1α [20]. Additionally, lactate induces histone lactylation in macrophages, an epigenetic modification that upregulates the expression of anti‐inflammatory genes [18].

Reprogramming TAMs from M2 to M1 phenotype can shift their function from tumor promoting to tumor suppressing. Thus, identifying novel molecules that modulate macrophage polarization may uncover new therapeutic targets for TAMs‐centric anti‐tumor immunotherapy [40]. Notably, in contrast to the attenuated M2 polarization observed in macrophages co‐cultured with PRDX1‐knockdown tumor cells, those co‐cultured with PRDX1‐overexpressing cells or treated directly with lactate exhibited a pronounced M2 phenotype. Intriguingly, lactate also upregulated PRDX1 expression in macrophages, suggesting a potential feedback loop. Whether lactate acts as a signal molecule or lactylation modification in manipulating the upregulation of PRDX1 requires further investigation. To decipher the reciprocal role of silencing PRDX1 in macrophages on CRC progression, we knocked down PRDX1 in macrophages and found that CM from BMDMs^PRDX1‐KO^ or RAW264.7^PRDX1‐KD^ significantly suppressed the growth and migration of CRC cells. Furthermore, co‐injection of BMDMs^PRDX1‐KO^ or RAW264.7^PRDX1‐KD^ cells inhibited syngeneic tumor growth by enhancing CD8^+^ T‐cell cytotoxicity and secretion of TNF‐α and GzmB. Future studies should identify the key signaling pathways and inflammatory cytokines alterations in PRDX1‐depleted TAMs to establish effective strategies for repolarizing TAMs toward an anti‐tumorigenic phenotype.

RNA‐sequencing results revealed the enrichment of cytokine and chemokine signaling pathways and activation of JAK‐STAT1 and NF‐κB signaling in RAW264.7^PRDX1‐KD^ cells. Subsequent inflammatory cytokine array analysis revealed significantly elevated levels of TNF‐α and IL‐1β, but reduced levels of IL‐6, in the CM of RAW264.7^PRDX1‐KD^ cells. This cytokine profile effectively suppressed the proliferation, migration, and colony formation of CRC cells in a co‐culture system. M1‐polarized macrophages are known to secrete high levels of IL‐1β and TNF‐α, which exert antiproliferative effects and can effectively kill tumor cells [41]. Consistent with our findings, M1 polarization is associated with inhibition of PI3K‐Akt signaling and activation of NF‐κB and JAK‐STAT1 pathways, leading to the secretion of inflammatory cytokines such as TNF‐α and IL‐1β [42]. In contrast, IL‐6, an inflammatory and immunosuppressive cytokine produced by both M1 and M2‐like macrophages, plays an important role in immune regulation, inflammation, and positively correlates with TNM stages of cancers [43]. In the TME, IL‐6/JAK/STAT3 signaling promotes tumor cell proliferation, angiogenesis, and metastasis, while strongly suppressing the antitumor immunity [44]. We found that PRDX1 deletion in macrophages reduced IL‐6 levels, thereby inhibiting tumor growth and enhancing antitumor immunity. Further studies are warranted to fully elucidate the underlying regulatory mechanism.

Collectively, our study unveils a novel role of PRDX1 in CRC progression and response to ICIs through crosstalk between CRC cells and TAMs. Specifically, knockdown of PRDX1 in CRC cells attenuated M2 macrophage polarization by reducing lactate secretion via the HIF‐1α/GLUT‐1 signaling pathway. These findings highlight the therapeutic potential of targeting PRDX1 in cancer cells to alleviate immunosuppression through suppression of M2 polarization. Reciprocally, PRDX1 ablation in macrophages restrains M2 polarization and enhances T‐cell‐mediated anti‐tumor immunity by promoting the secretion of inflammatory factors (IL‐1β and TNFα) via activation of JAK‐STAT1/NF‐κB signaling pathways. These results underscore the therapeutic potential of targeting PRDX1 to reverse immunosuppression and enhance anti‐tumor immunity in CRC.

Despite the insights gained from our study, several limitations should be acknowledged. First, the upstream mechanisms that link PRDX1 ablation in macrophages to the activation of JAK‐STAT1/NF‐κB signaling and M1 polarization remain incompletely defined. Second, our findings regarding the PRDX1‐CUL2‐HIF‐1α axis in cancer cells are primarily based on in vitro evidence, and the in vivo relevance of the PRDX1‐CUL2 interaction warrants further validation. Third, although we observed that lactate can upregulate PRDX1 in macrophages, the existence and functional impact of this potential feedback loop require more direct experimental evidence. Finally, it should be noted that these findings primarily derive from preclinical models, and further validation in clinical settings is essential to assess their translational relevance.

In conclusion, the molecular chaperone activity of PRDX1 to bind to CUL2 was at least as important as the peroxidase activity, providing new insights into the role of PRDX1 for drug design to inhibit CRC progression by modulating anti‐tumor immunity.

## 4 Materials and Methods

### 4.1 Cell Culture and Lentivirus Infection

Murine CRC cell lines MC38 and CT26, human CRC cell lines HCT116 and SW620, and murine macrophage RAW264.7 were originally obtained from the American Type Culture Collection (ATCC) and cultured according to the manufacturer's instructions. Cell line authenticity was verified using short tandem repeat (STR) profiling and confirmed to be mycoplasma free.

Human CRC cells were transduced with PRDX1‐overexpressing lentiviral particles (PRDX1‐OE) and the vector control (pHBLV‐CMV‐MCS‐3FLAG‐EF1‐ZsGreen‐T2A‐PURO) (HANBIO, Shanghai, China). To knockdown PRDX1, human CRC cells were infected with shPRDX1 lentivirus (PRDX1‐KD) and the vector control GV248 (GeneChem, Shanghai, China). RAW264.7 cells were infected with murine shPRDX1 lentivirus (PRDX1‐KD) and vector control (pHBLV‐U6‐MCS‐CMV‐ZsGreen‐PGK‐PURO) (HANBIO, Shanghai, China). The cells were then treated with puromycin (10 µg/mL) to establish stable cell lines.

### 4.2 Preparation of Murine BMDMs and Conditioned Media

Murine BMDMs were isolated from the femurs and tibias of wild‐type C57BL/6J mice and *PRDX1*‐KO) mice aged at 6–8 weeks using previously described methods [45]. After removal of erythrocytes with RBC lysis buffer (Invitrogen, USA), cells were cultured in BMDM differentiation medium (high glucose DMEM, 10% heat‐inactivated FBS supplemented with 100 U/mL penicillin, 0.1 mg/mL streptomycin, and 10 ng/mL M‐CSF) for 5–7 days. To induce M2 polarization, BMDMs were stimulated with IL‐4 (20 ng/mL).

To prepare CM, when the cell density reached approximately 80%, the cell medium was replaced with serum‐free medium for 24 h before harvesting. The CM was then centrifuged at 500 g for 5 min, filtrated through 0.22 µm filters, and stored at −80°C.

### 4.3 Clinical Specimens

Human CRC and paired adjacent normal colonic tissue specimens were collected from CRC patients undergoing surgical resection prior to chemotherapy at Xuanwu Hospital of Capital Medical University (Beijing, China). The MSS and MSI‐H status of the tissue specimens (*n* = 54) were evaluated by pathological analysis and IHC staining. All procedures were conducted in accordance with the Declaration of Helsinki and approved by the Institutional Review Board of Capital Medical University (Z2023SY009). The clinical parameters of CRC patients are listed in Table S1.

### 4.4 RNA Sequencing

Total RNA was extracted from RAW264.7 cells infected with PRDX1 shRNA lentivirus (PRDX1‐KD) and negative control lentivirus (CON‐KD) using Trizol reagent (Invitrogen, USA). Similarly, RNA was extracted from AOM/DSS‐induced colonic cancer tissues from WT and *PRDX1*‐KO mice. RNA sequencing was conducted on the Hiseq 4000 platform (Beijing Genomics Institute, Wuhan, China) as previously described [46]. The raw transcriptome sequencing data have been deposited in the sequence read archive (SRA) with BioProject accession numbers PRJNA1110181 and PRJNA1110607, respectively.

### 4.5 RT‐qPCR Analysis

RT‐qPCR analysis was performed on 7500 Fast Real‐Time PCR System (Applied Biosystems, USA) following established protocols [47]. Primer sequences for macrophage polarization markers, including *NOS2*, *Arg‐1*, *TNFɑ*, *CD206* and *IL‐10*, have been described previously [48]. The additional primer sequences are listed in Table S2.

### 4.6 Western Blotting

Western blot analysis was conducted following established methods [47]. Primary antibodies are listed in Table S3. Chemiluminescence signals were captured using the Fluorchem FC3 imaging system (ProteinSimple, USA) and quantified with the NIH ImageJ software.

### 4.7 Immunohistochemistry

IHC staining was performed on 5‐µm‐thick formalin‐fixed, paraffin‐embedded (FFPE) colorectal tissue sections following standard procedures as previously described [47]. Tissue sections were incubated with primary antibodies (Table S3) in accordance with the manufacturers’ instructions. Stained sections were scanned using a digital slide scanning system (Pannoramic Scan, 3DHISTECH Ltd) and the mean density (IOD/area) was semi‐quantified using image‐pro plus 6 software (IPP, USA).

### 4.8 mIF Staining

mIF staining of tumor tissue from mice was performed following standard protocols. Briefly, FFPE sections were deparaffinized, rehydrated, and subjected to antigen retrieval. The samples were stained for mIF using the following primary antibodies: CD4 (1:1000, ab183685, Abcam), CD206 (1:1000, 24595, Cell Signaling Technology), and CD8 (1:2000, ab217344, Abcam). The TG 6‐colour Manual TSA Kit (TGFP6100, TissueGnostics) was used according to the manufacturer's instructions. Nuclei were counterstained with DAPI (1:200, C1006, Beyotime) for 5 min, and slides were mounted using anti‐fade fluorescence mounting medium. Images were acquired using the TissueFAXS imaging system (TissueGnostics).

### 4.9 Co‐IP Assay

HEK293 cells were transfected with Flag‐WT‐PRDX1 and Flag‐Cys83Ser‐PRDX1 together with HA‐Cullin‐2 plasmids using Lipofectamine 2000 transfection reagent (Invitrogen, USA). Co‐IP was performed using BeaverBeads protein A/G Immunoprecipitation Kit (BEAVER, China) following established methods [46]. Antibodies used for IP are listed in Table S3.

### 4.10 Ubiquitination Assay

HCT116^PRDX1‐KD^ and SW620^PRDX1‐KD^ cells, along with their respective controls, were treated with 20 µM MG132 for 6 h. The ubiquitination of HIF‐1α was assessed by IP using an anti‐HIF‐1α antibody, followed by detection of the immune complex with an anti‐ubiquitin antibody (Table S3) and protein A/G magnetic beads (BEAVER, China).

### 4.11 Flow Cytometry

Mouse TAMs and T cells were isolated from subcutaneous tumor tissues using a previously described method [49]. Briefly, tumor tissues were minced and enzymatically digested in RPMI1640 medium containing 1 mg/mL collagenase IV, 0.2 mg/mL hyaluronidase, and 20 U/mL DNAase IV at 37°C for 2 h with rotation. The cell suspension was then filtered through a 40‐µm nylon mesh and purified on 40% and 70% Percoll gradients. TAMs were stained with surface markers FITC‐CD11b, PerCP/Cy5.5‐F4/80, PE‐CD86, followed by intracellular staining with APC‐CD206 antibody after incubation with Fixation and Permeabilization Solution (BioLegend, USA). T cells were surface stained with BV510‐CD3, AF647‐CD4, and AF700‐CD8a antibodies to determine subset percentages. To assess the functions of CD8^+^ T cells, cell suspensions were cultured in RPMI 1640 medium supplemented with 10% FBS, 100 U/mL penicillin, and 100 mg/mL streptomycin, and were activated by Cell Activation Cocktail (PMA/ionomycin) with Brefeldin A and Monensin to block trafficking for 6 h before intracellular staining with AF488‐IFN‐γ, PE‐TNF‐α, PE‐Cy7‐GzmB, and BV421‐PD‐1 antibodies (BioLegend, USA) for 1 h on ice in the dark. The antibodies are listed in Table S4. Data were acquired on a BD FACSymphony flow cytometer and analyzed using TreeStar Flowjo software.

### 4.12 In Vitro Phagocytosis Assays

MC38 and CT26 cells were labeled with CFSE fluorescence using the CFDA‐SE Cell Proliferation and Cell Tracking Kit (Yeasen Biotechnology, Shanghai, China). BMDMs isolated from WT and PRDX1‐KO mice were seeded into 12‐well plates and co‐cultured with CFSE‐labeled murine MC38 and CT26 cells for 6 h. The cells were then trypsinized and stained with PerCP/Cy5.5‐F4/80 antibody for 40 min on ice, followed by flow cytometry analysis. Phagocytosis efficiency was quantified by flow cytometry as the percentage of CFSE‐positive cells within the F4/80^+^ macrophage population.

### 4.13 Cytokine Arrays

The cytokine secretion profiles of macrophages (BMDMs and RAW264.7 cells) were analyzed using the G‐Series Mouse Cytokine Array 1 (#GSM‐CYT‐1, RayBiotech, USA) in triplicate following the manufacturer's instructions and previously described methods [48].

### 4.14 Tumor Cell Proliferation and Migration Assay

CRC cell proliferation was measured using the MTT assay. Briefly, CRC cells (2 × 10^3^/well) were seeded in 96‐well plates, serum‐starved overnight, and treated with CM from BMDM^WT^ and BMDM^PRDX1‐KO^ as well as RAW264.7^CON‐KD^ and RAW264.7^PRDX1‐KD^ cells for indicated time points. MTT reagent was added, and formazan absorbance was measured at 570 nm using a spectrophotometer.

Cell migration was assessed using Transwell Boyden chambers (Corning, USA). CRC cells (2 × 10⁴/well) in serum‐free medium were seeded in the upper chamber, while the lower chamber contained BMDM^WT^ and BMDM^PRDX1‐KO^ as well as RAW264.7^CON‐KD^ and RAW264.7^PRDX1‐KD^ cells. After 24 h, migrated cells were fixed, stained with 0.2% crystal violet, and quantified by counting three random fields under a light microscope.

### 4.15 Colony Formation

For the colony formation assay, CRC cells were seeded in six‐well plates (800 cells/well). These cells were incubated with CM from BMDMs and RAW264.7 cells for 14 days. Colonies were then fixed and stained with 0.2% crystal violet solution (Beyotime, C0121) following the manufacturer's protocol.

### 4.16 L‐Lactate Assay

L‐Lactate levels were measured using the L‐Lactate Assay Kit (Jiangcheng, Nanjing, China) following the manufacturer's instructions. Briefly, CRC cells were lysed using an ultrasonic processor (VCX 130, Sonics& Materials, Inc.), reacted with enzyme working buffer to generate a purple product. Absorbance was measured at 530 nm using a spectrophotometer (Molecular Devices, CA, USA).

### 4.17 Silver Staining and Immunoprecipitation‐Mass Spectrometry

IP was performed using the BeaverBeads protein A/G Immunoprecipitation Kit (BEAVER, China). Briefly, cell lysate from Flag‐tagged HCT116^PRDX1‐OE^ and HCT116^CON‐OE^ was immunoprecipitated with anti‐Flag antibody or normal rabbit IgG. The proteins interacting with Flag‐PRDX1 were resolved by SDS‐PAGE and visualized using a Silver Staining Kit (Thermo Scientific, USA) according to the manufacturer's instructions. Polyacrylamide gels were fixed, sensitized, and developed until protein bands appeared, followed by the stop solution. The differential protein bands were excised and analyzed by liquid chromatography‐tandem mass spectrometry (LC‐MS/MS, AB SCIEX) using ProteinPilot software for protein identification.

### 4.18 Mice and Tumor Models

Balb/c and C57BL/6J mice, 6–8 weeks old, were obtained from Vital River Laboratories (Beijing, China). The genotyping of PRDX1‐KO mice was described previously [11]. All mice were housed under specific pathogen‐free conditions, and experiments were approved by the Animal Welfare Committee of Capital Medical University (ethical number: AEEI‐2021‐194).

For subcutaneous tumor models, MC38 cells (1 × 10^6^) were mixed with BMDMs from WT and PRDX1‐KO mice at a 3:1 ratio and then injected subcutaneously into the right flank of syngeneic C57BL/6J mice that were depleted of macrophages using clodronate liposomes (1 mg/mice/day i.p. for 2 days). Similarly, CT26 cells were mixed with RAW264.7^CON‐KD^ and RAW264.7^PRDX1‐KD^ cells (3:1) and inoculated into Balb/c mice. After implantation for 1 week, tumor volumes were measured every other day using calipers (volume = length × width^2^/2). Tumor‐bearing mice were randomized into four groups and injected intraperitoneally twice a week with anti‐PD‐1 antibody (10 mg/kg, BioXCell, cat. BE0146) or the corresponding IgG control (BioXCell, cat. BE0089) for 2 weeks. Mice were euthanized and tumors were collected for further analysis.

The AOM/DSS colitis‐associated colonic adenocarcinomas model was established using the previously reported method [50]. Mice were sacrificed and colonic tumors were counted.

### 4.19 Bioinformatics

The expression of PRDX1 in Pan‐cancers between tumor tissues and adjacent normal tissues was analyzed by Sangerbox (http://vip.Sangerbox.com/home.html) using UCSC database. TIMER2.0 (http://timer.comp‐genomics.org) was used to determine the correlation of PRDX1 expression with purity and infiltration levels of T cells and macrophages.

Single‐cell sequencing datasets (GSE166555 and GSE205506) were used to analyze the expression level of PRDX1 in different cell clusters. We normalized and scaled the data using Seurat (v4.3.0) [51], then used the “vst” algorithm to select the top 2000 variable genes using the “FindVariable” function and performed principal component analysis (PCA), and removed batch effects between the data using the R package Harmony [52]. The first 20 principal components (PCs) and a resolution of 0.5 were used to classify all cells, which were then visualized using umap [53]. Cellular annotation was performed by the expression levels of the following cellular markers: macrophages (CD68, CLEC7A, and CD163), T cells (CD3D and CD3E), B cells (CD79A and JCHAIN), epithelial cells (KRT8, KRT18, and EPCAM), fibroblasts (COL1A1 and COL1A2), and endothelial cells (PECAM and CLDN5). Wilcoxon rank‐sum test was used to identify marker genes in all clusters. The expression levels of *PRDX1* gene in different groups of patients were statistically analyzed using the R package ggpubr and visualized using violin plots.

### 4.20 Statistical Analysis

Data are expressed as mean ± SD from triplicate experiments. Statistical analysis was performed using GraphPad Prism 8, with group comparisons analyzed by Student's *t*‐test or one‐way ANOVA. A *p*‐value < 0.05 (two‐tailed) was considered statistically significant.

## Author Contributions

X.Y. conceived the study, designed the research, and drafted the manuscript. Y.Q.‐S. carried out the western blot, RT‐qPCR analysis, animal experiments, and drafted the manuscript. J.H. participated in the immunofluorescence assay and flow cytometry; X.W. collected the clinical specimens; N.Y., X.L., and Y.J.‐S. participated in the cell culture; J.Q. performed the bioinformation analysis; and X.X. helped in the flow cytometry. All authors have read and approved the final manuscript.

## Funding

This study was supported by the National Natural Science Foundation of China (82473944 and 82073876) and Beijing Natural Science Foundation (7202012).

## Ethics Statement

This study was approved by the Ethics Committee of Capital Medical University. Informed consent was obtained from all the patients and patient data have been made anonymous. All procedures were approved by the Institutional Review Board of Capital Medical University (Z2023SY009). All animal experiments were approved by the Institutional Animal Care and Use Committee of Capital Medical University. The ethics number was AEEI‐2021‐194.

## Conflicts of Interest

The authors declare no conflicts of interest.

## Supporting information




**Fig. S1**: Expression and immunoregulatory role of PRDX1 across cancers. (A) Pan‐cancer analysis of PRDX1 expression in tumor tissues compared with adjacent normal tissues based on data from the UCSC database using Sangerbox. (B) Correlation between PRDX1 expression and infiltration levels of CD4⁺ T cells, CD8⁺ T cells, and M2 macrophages in CRC was analyzed using TIMER 2.0. (C) GSEA showing enrichment of antigen processing and presentation pathways associated with PRDX1 expression.
**Fig. S2**: PRDX1 modulates macrophage polarization and HIF1ɑ/GLUT1 axis. (A) Western blot analysis of PRDX1 expression in CT26^CON‐KD^ and CT26^PRDX1‐KD^ cells. β‐actin was used as a loading control. (B) Representative bright‐field images of BMDMs from WT and PRDX1‐KO mice. (C) Flow cytometric identification of BMDMs based on CD11b⁺ and F4/80⁺ expression. (D) RT‐qPCR analysis of the mRNA levels of macrophage M2 polarization markers in RAW264.7 cells after co‐culture with conditioned media (CM) from CT26^PRDX1‐OE^ cells. Data are presented as mean ± SD. **p < 0.01, ***p < 0.001, *n* = 4. (E) RT‐qPCR analysis of PRDX1 mRNA levels in HCT116 and SW620 cells infected with PRDX1‐KD or control lentivirus. (F, G) Western blot analysis of the expression of glycolysis‐related genes (HK1, PKM1/2, LDHA, PFKP) in SW620^PRDX1‐KD^ and HCT116 ^PRDX1‐KD^ cells compared to their respective controls. Densitometric quantification of protein levels is shown. (H) Lactate levels were determined in the SW620^PRDX1‐KD^ cells transfected with Flag‐GLUT1 plasmid, and in CT26^PRDX1‐KD^ or CT26^PRDX1‐OE^ cells versus their respective controls. (I) RT‐qPCR analysis of PRDX1 mRNA levels in RAW264.7 cells treated with indicated concentrations of lactate for 24 h. Data are presented as mean ± SD. *p < 0.05, **p < 0.01, *n* = 3.
**Fig. S3**: PRDX1 regulates HIF‐1α stability and macrophage polarization in colorectal cancer. (A) Western blot analysis of HIF1ɑ, GLUT1 levels in SW620^PRDX1‐KD^ cells transfected with or without Flag‐HIF‐1α plasmid. β‐actin was used as a loading control. (B) Western blot analysis of the expression of HIF1ɑ, GLUT1 protein in CT26^PRDX1‐KD^ or CT26^PRDX1‐OE^ cells compared to their respective controls. Data are presented as mean ± SD. *p < 0.05, **p < 0.01, ***p < 0.001, *n* = 3. (C) CHX chase assay was performed to evaluate the degradation of HIF‐1α in SW480^PRDX1‐OE^ cells compared to SW480^CON‐OE^ cells. (D) Analysis of PRDX1 expression in macrophages from CRC and normal tissue based on the GSE 166555 dataset. (E) Flow cytometry analysis of the polarization phenotype of BMDMs^WT^ and BMDMs^PRDX1‐KO^ by F4/80‐PerCp‐Cy5.5 and CD206‐APC staining. Data are presented as mean ± SD, * *P* <0.05, n = 3.
**Fig. S4**: Transcriptomic analysis in PRDX1‐deficient macrophages. (A) Volcano plot showing differentially expressed genes between RAW264.7^PRDX1‐KD^ and RAW264.7^CON‐KD^ cells. (B) GSEA of all differentially expressed genes between RAW264.7^CON‐KD^ and RAW264.7^PRDX1‐KD^ cells. (C) Heatmap of differentially expressed cytokines between BMDMs^PRDX1‐KO^ and BMDMs^WT^. (D) Flow cytometry analysis of percentages of splenic macrophages from mice injected intraperitoneally with clodronate liposomes (SP‐CL) compared to control group (SP‐CON).
**Fig. S5**: PRDX1 deficiency in macrophages inhibits tumor growth and modulates immune profiles in a MC38 syngeneic model. (A) Representative images of syngeneic tumors in C57BL/6J mice inoculated with MC38 cells and BMDMs^WT^ or BMDMs^PRDX1‐KO^ respectively (3:1 ratio). The tumor weight was measured and data are presented as mean ± SD, **p < 0.01, *n* = 4. (B, C) mIF analysis of CD206 (purple), CD8 (yellow) and CD4 (green) expression in tumor tissues. Scale bar = 100 µm. (D, E) Flow cytometry analysis of the expression of CD206‐APC and CD86‐PE in CD11b^+^/F4/80^+^ macrophages, and IFN‐γ‐AF488, TNF‐α‐PE, GzmB‐PE‐Cy7 and PD‐1‐BV421 expression in CD8^+^ T cells. Data are presented as mean ± SD. * *p* < 0.05, ** *p* < 0.01, n = 3.
**Table S1**: Demographic information of colorectal cancer patients.
**Table S2**: The sequences of primers for RT‐qPCR.
**Table S3**: The antibodies and application.
**Table S4**: Antibodies for identification of the immunocytes using flow cytometry.
**Table S5**: The significantly upregulated genes in CRC tissues of PRDX1‐KO v.s. WT mice.
**Table S6**: The significantly differential genes in RAW264.7^PRDX1‐KD^ v.s. RAW264.7^CON‐KD^ (listed in part)
**Table S7**: The levels of cytokines in conditioned medium of RAW264.7^PRDX1‐KD^ v.s. RAW264.7^CON‐KD^
**Table S8**: The levels of cytokines in conditioned medium of BMDMs^PRDX1‐KO^ v.s. BMDMs^WT^

## Data Availability

The data that support the findings of this study are available from the corresponding author upon reasonable request. Additional data are available as the Supporting Information.
