# Supplementary material for: Targeted Deletion of Peroxiredoxin 1 Enhances Anti‐Tumor Immunity in Colorectal Cancer by Reprogramming the Immunosuppressive Tumor‐Associated Macrophages
Source: MedComm (2020). 2025 Nov 24;6(12):e70495. doi: 10.1002/mco2.70495 (PMC12644249; doi:10.1002/mco2.70495)
Supplement: Supplementary file 1 — Fig. S1: Expression and immunoregulatory role of PRDX1 across cancers. (A) Pan‐cancer analysis of PRDX1 expression in tumor tissues compared with adjacent normal tissues based on data from the UCSC database using Sangerbox. (B) Correlation between PRDX1 expression and infiltration levels of CD4⁺ T cells, CD8⁺ T cells, and M2 macrophages in CRC was analyzed using TIMER 2.0. (C) GSEA showing enrichment of antigen processing and presentation pathways associated with PRDX1 expression. Fig. S2: PRDX1 modulates macrophage polarization and HIF1ɑ/GLUT1 axis. (A) Western blot analysis of PRDX1 expression in CT26CON‐KD and CT26PRDX1‐KD cells. β‐actin was used as a loading control. (B) Representative bright‐field images of BMDMs from WT and PRDX1‐KO mice. (C) Flow cytometric identification of BMDMs based on CD11b⁺ and F4/80⁺ expression. (D) RT‐qPCR analysis of the mRNA levels of macrophage M2 polarization markers in RAW264.7 cells after co‐culture with conditioned media (CM) from CT26PRDX1‐OE cells. Data are presented as mean ± SD. **p < 0.01, ***p < 0.001, n = 4. (E) RT‐qPCR analysis of PRDX1 mRNA levels in HCT116 and SW620 cells infected with PRDX1‐KD or control lentivirus. (F, G) Western blot analysis of the expression of glycolysis‐related genes (HK1, PKM1/2, LDHA, PFKP) in SW620PRDX1‐KD and HCT116 PRDX1‐KD cells compared to their respective controls. Densitometric quantification of protein levels is shown. (H) Lactate levels were determined in the SW620PRDX1‐KD cells transfected with Flag‐GLUT1 plasmid, and in CT26PRDX1‐KD or CT26PRDX1‐OE cells versus their respective controls. (I) RT‐qPCR analysis of PRDX1 mRNA levels in RAW264.7 cells treated with indicated concentrations of lactate for 24 h. Data are presented as mean ± SD. *p < 0.05, **p < 0.01, n = 3. Fig. S3: PRDX1 regulates HIF‐1α stability and macrophage polarization in colorectal cancer. (A) Western blot analysis of HIF1ɑ, GLUT1 levels in SW620PRDX1‐KD cells transfected with or without Flag‐HIF‐1α plasmid. β‐acti [file MCO2-6-e70495-s001.docx]

**Supporting information**

Targeted deletion of Peroxiredoxin 1 enhances anti-tumor immunity in colorectal cancer by reprogramming the immunosuppressive tumor-associated macrophages

**Authors**

Yuqi Sun^1#^, Jinli Han^1#^, Nianhua Yu^1^, Jinglin Qin^2^, Xiaohui Wang^3^, Xi Li^1^, Yujia Song^1^, Xiaoxue Xu^4^, Xinfeng Yu^1*^.

**Affiliations**

^1^ Department of Pharmacology, School of Basic Medical Sciences, Capital Medical University, Beijing, China.

^2^ Department of Neurobiology, School of Basic Medical Sciences, Beijing Key Laboratory of Neural Regeneration and Repair, Capital Medical University, Beijing 100069, China.

^3^ Department of General Surgery, Xuanwu Hospital, Capital Medical University, Beijing, China.

^4^ Department of Core Facility Center, Capital Medical University, Beijing, China.

^#^ These authors contribute equally to the work.

^*^ Correspondence should be addressed to Xinfeng Yu (xyu@ccmu.edu.cn)

**Supplementary Figures and Tables**


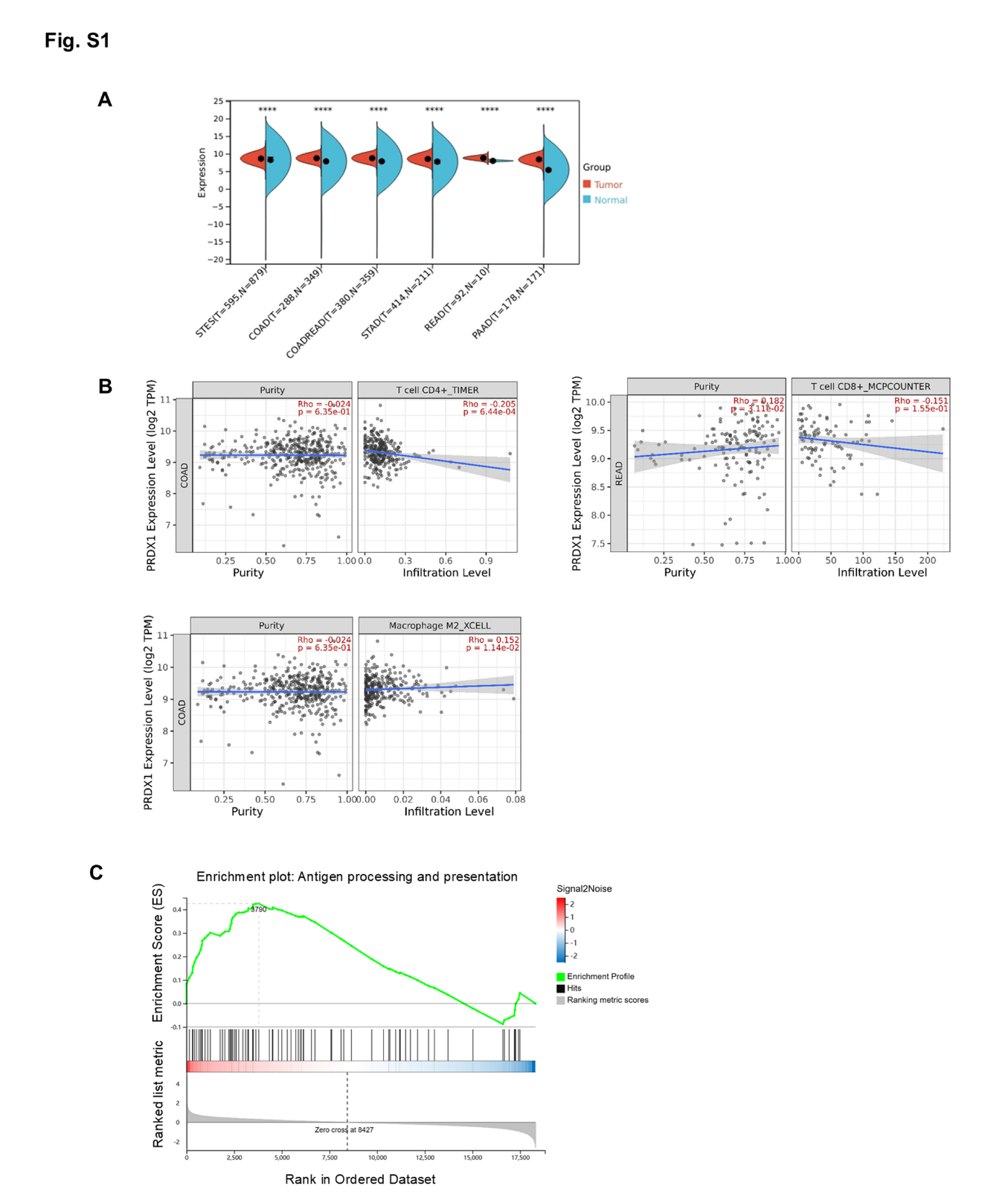


**Fig. S1 Expression and immunoregulatory role of PRDX1 across cancers.** (A) Pan-cancer analysis of PRDX1 expression in tumor tissues compared with adjacent normal tissues based on data from the UCSC database using Sangerbox. (B) Correlation between PRDX1 expression and infiltration levels of CD4⁺ T cells, CD8⁺ T cells, and M2 macrophages in CRC was analyzed using TIMER 2.0. (C) GSEA showing enrichment of antigen processing and presentation pathways associated with PRDX1 expression.


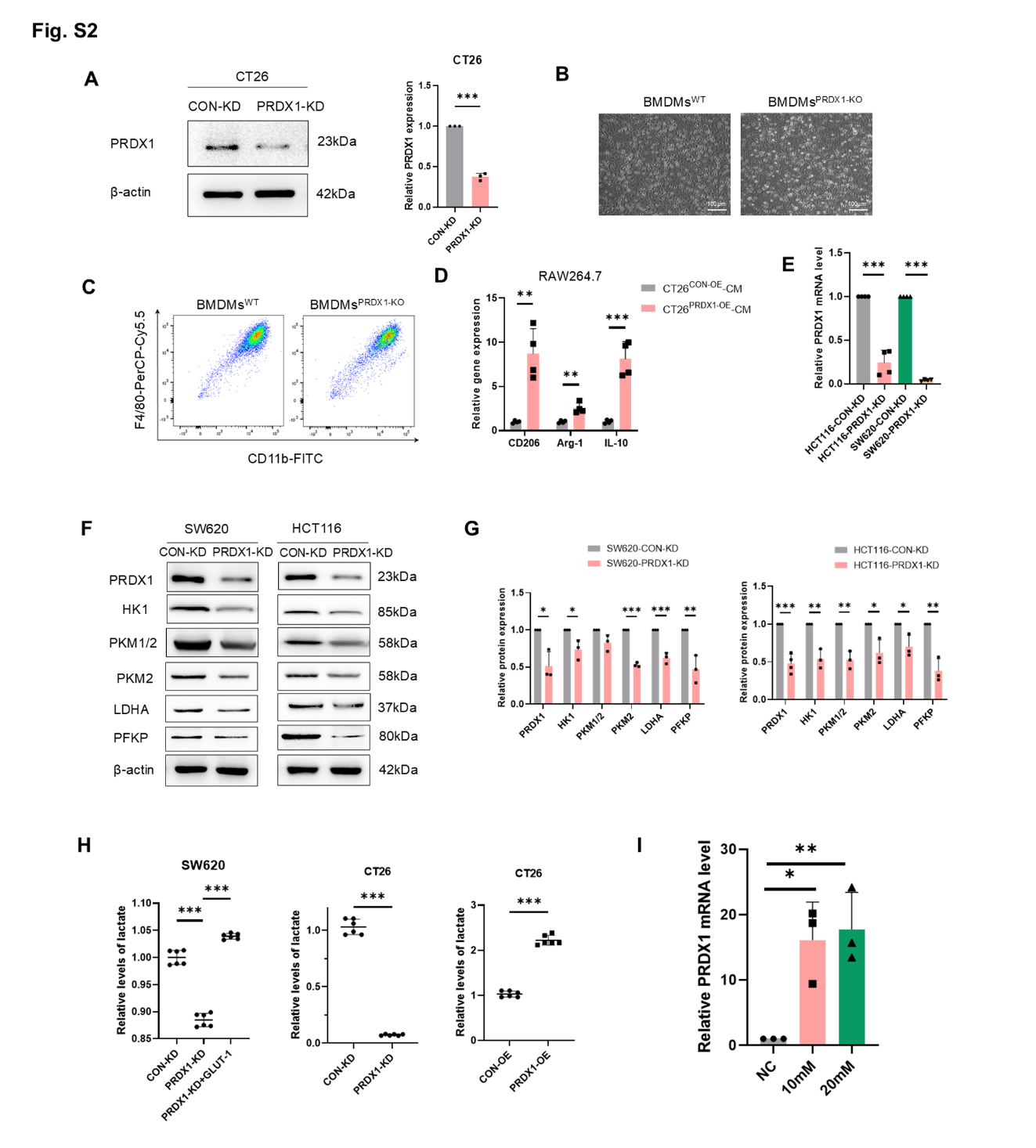


**Fig. S2 PRDX1 modulates macrophage polarization and HIF1ɑ/GLUT1 axis.** (A) Western blot analysis of PRDX1 expression in CT26^CON-KD^ and CT26^PRDX1-KD^ cells. β-actin was used as a loading control. (B) Representative bright-field images of BMDMs from WT and PRDX1-KO mice. (C) Flow cytometric identification of BMDMs based on CD11b⁺ and F4/80⁺ expression. (D) RT-qPCR analysis of the mRNA levels of macrophage M2 polarization markers in RAW264.7 cells after co-culture with conditioned media (CM) from CT26^PRDX1-OE^ cells. Data are presented as mean ± SD. ***P* < 0.01, ****P* < 0.001, *n* = 4. (E) RT-qPCR analysis of PRDX1 mRNA levels in HCT116 and SW620 cells infected with PRDX1-KD or control lentivirus. (F, G) Western blot analysis of the expression of glycolysis-related genes (HK1, PKM1/2, LDHA, PFKP) in SW620^PRDX1-KD^ and HCT116 ^PRDX1-KD^ cells compared to their respective controls. Densitometric quantification of protein levels is shown. (H) Lactate levels were determined in the SW620^PRDX1-KD^ cells transfected with Flag-GLUT1 plasmid, and in CT26^PRDX1-KD^ or CT26^PRDX1-OE^ cells versus their respective controls. (I) RT-qPCR analysis of PRDX1 mRNA levels in RAW264.7 cells treated with indicated concentrations of lactate for 24 h. Data are presented as mean ± SD. **P* < 0.05, ***P* < 0.01, *n* = 3.


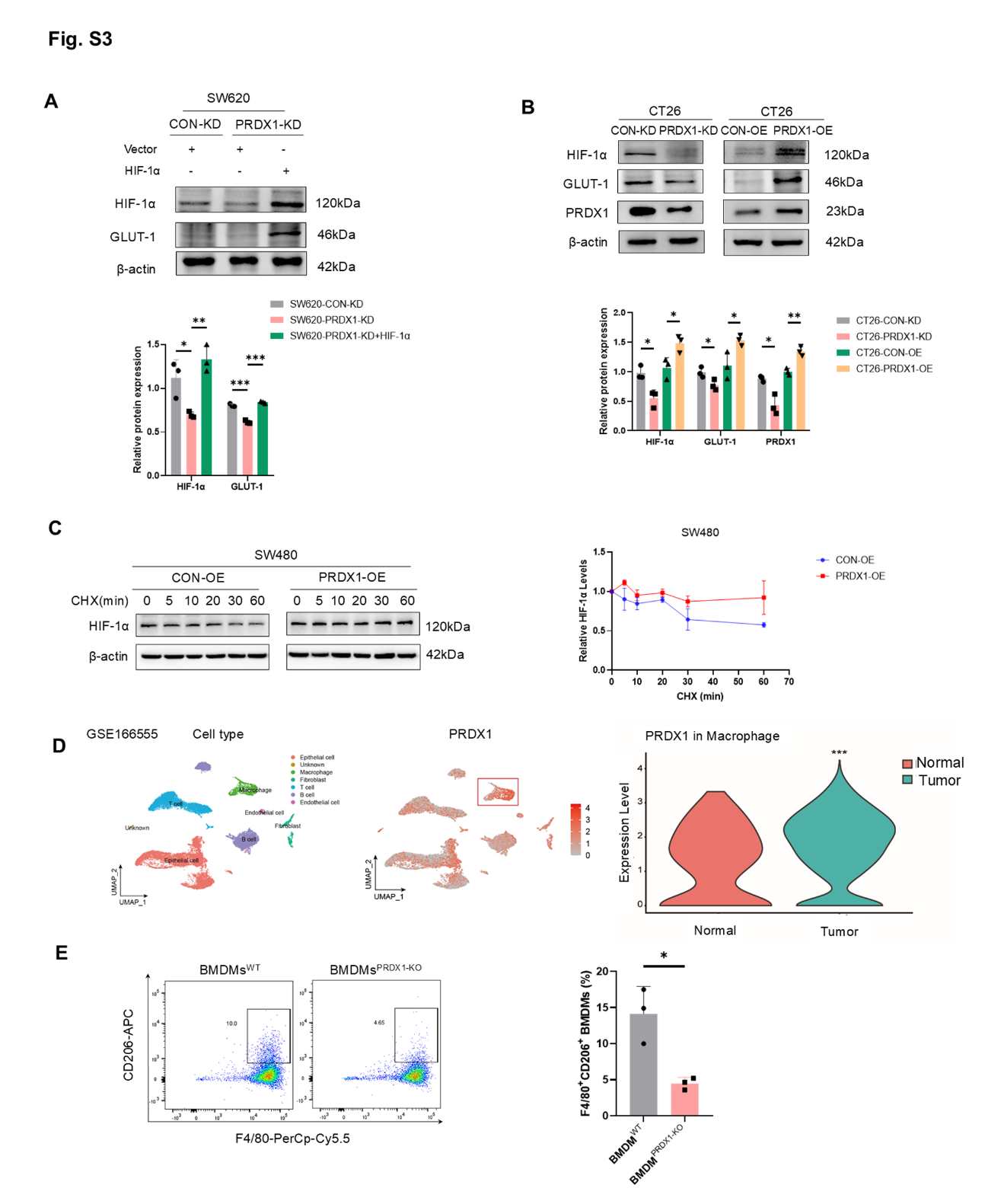


**Fig. S3 PRDX1 regulates HIF-1α stability and macrophage polarization in colorectal cancer.** (A) Western blot analysis of HIF1ɑ, GLUT1 levels in SW620^PRDX1-KD^ cells transfected with or without Flag-HIF-1α plasmid. β-actin was used as a loading control. (B) Western blot analysis of the expression of HIF1ɑ, GLUT1 protein in CT26^PRDX1-KD^ or CT26^PRDX1-OE^ cells compared to their respective controls. Data are presented as mean ± SD. **P* < 0.05, ***P* < 0.01, ****P* < 0.001, *n* = 3. (C) CHX chase assay was performed to evaluate the degradation of HIF-1α in SW480^PRDX1-OE^ cells compared to SW480^CON-OE^ cells. (D) Analysis of PRDX1 expression in macrophages from CRC and normal tissue based on the GSE 166555 dataset. (E) Flow cytometry analysis of the polarization phenotype of BMDMs^WT^ and BMDMs^PRDX1-KO^ by F4/80-PerCp-Cy5.5 and CD206-APC staining. Data are presented as mean ± SD, * *P* <0.05, n = 3.


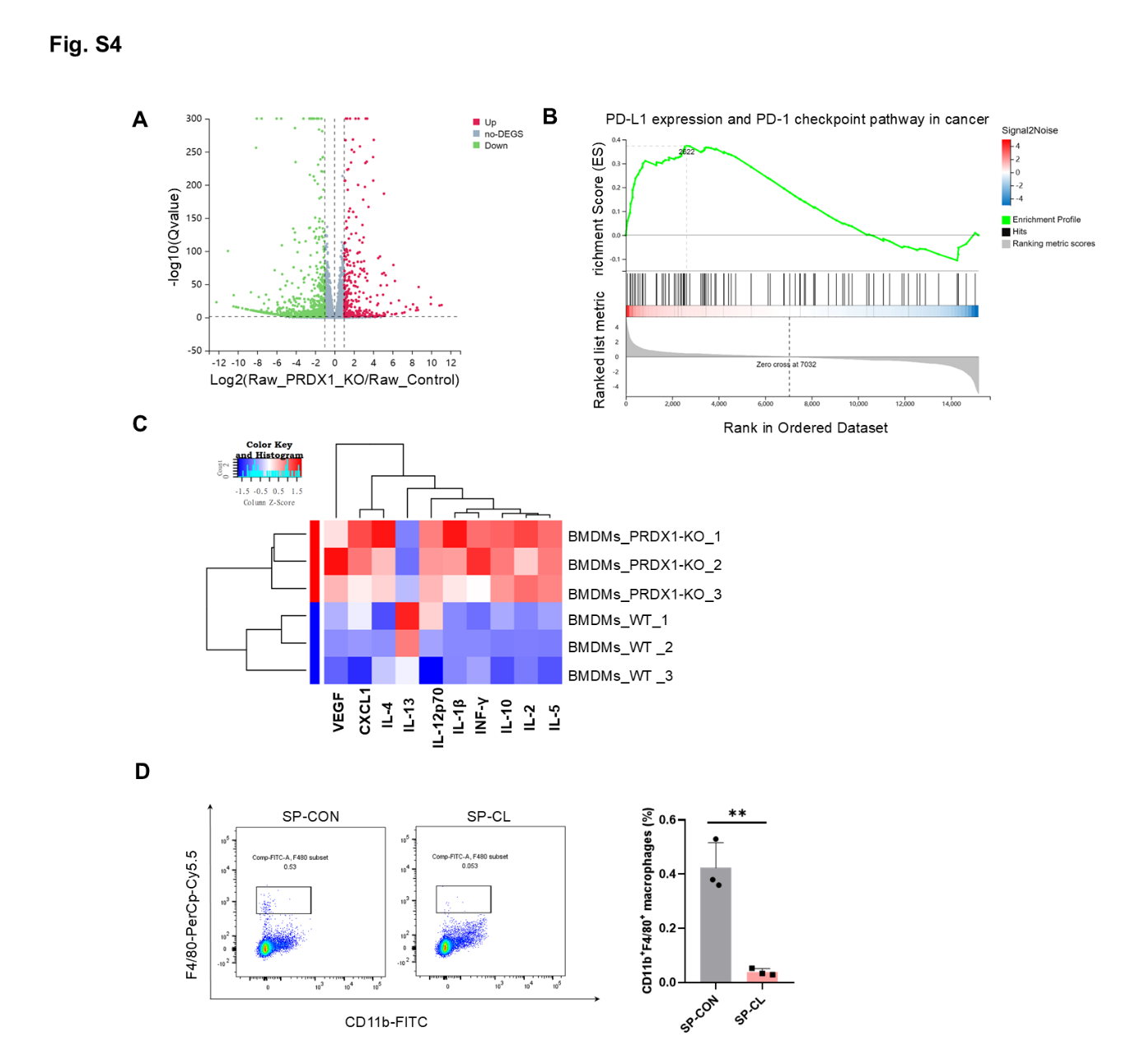


**Fig. S4 Transcriptomic analysis in PRDX1-deficient macrophages.** (A) Volcano plot showing differentially expressed genes between RAW264.7^PRDX1-KD^ and RAW264.7^CON-KD^ cells. (B) GSEA of all differentially expressed genes between RAW264.7^CON-KD^ and RAW264.7^PRDX1-KD^ cells. (C) Heatmap of differentially expressed cytokines between BMDMs^PRDX1-KO^ and BMDMs^WT^. (D) Flow cytometry analysis of percentages of splenic macrophages from mice injected intraperitoneally with clodronate liposomes (SP-CL) compared to control group (SP-CON).


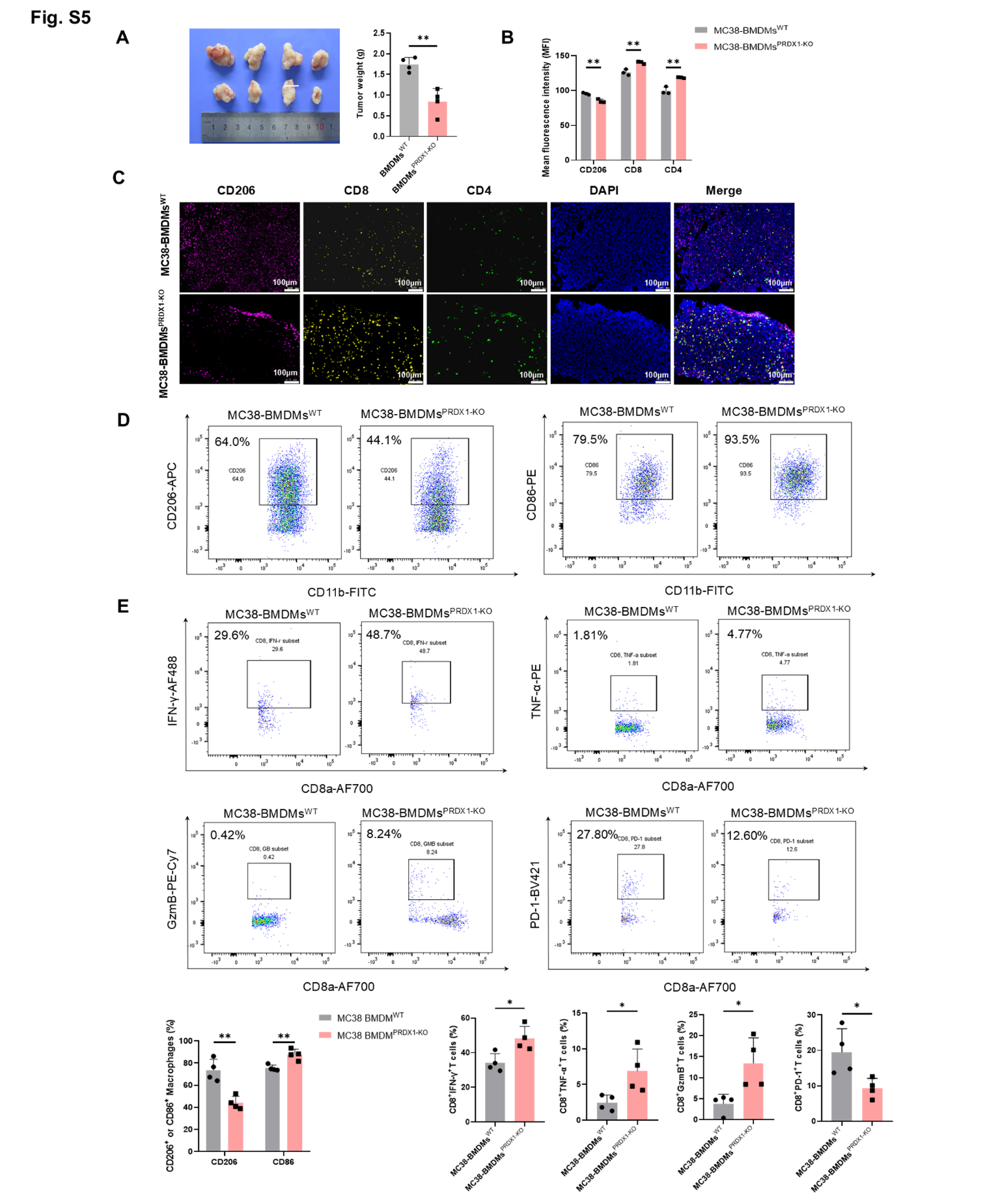


**Fig. S5** **PRDX1 deficiency in macrophages inhibits tumor growth and modulates immune profiles in a MC38 syngeneic model.** (A) Representative images of syngeneic tumors in C57BL/6J mice inoculated with MC38 cells and BMDMs^WT^ or BMDMs^PRDX1-KO^ respectively (3:1 ratio). The tumor weight was measured and data are presented as mean ± SD, ***P* < 0.01, *n* = 4. (B, C) mIF analysis of CD206 (purple), CD8 (yellow) and CD4 (green) expression in tumor tissues. Scale bar = 100 μm. (D, E) Flow cytometry analysis of the expression of CD206-APC and CD86-PE in CD11b^+^/F4/80^+^ macrophages, and IFN-γ-AF488, TNF-α-PE, GzmB-PE-Cy7 and PD-1-BV421 expression in CD8^+^ T cells. Data are presented as mean ± SD. * *P* < 0.05, ** *P* < 0.01, n = 3.

**Table S1. Demographic information of colorectal cancer patients**

|  | Variables | Number | Percentage（%） |
| --- | --- | --- | --- |
| Age | <=70 | 36 | 67 |
|  | >70 | 18 | 33 |
| Gender | Male | 31 | 57 |
|  | Female | 23 | 43 |
| Location | Colon | 39 | 72 |
|  | Rectum | 15 | 28 |
| Immunophenotyping | MSS | 45 | 83 |
|  | MSI | 9 | 17 |

**Table S2. The sequences of primers for RT-qPCR**

| Gene | ﻿Primer Sequence (5′ to 3′) | Species |
| --- | --- | --- |
| PRDX1 | F: caactgccaagtgattggtg  R: tgatctgccgaagaataccc | *Homo sapiens/Mus musculus* |
| IL-6 | F:ccggagaggagacttcacag  R:cagaattgccattgcacaac | *Mus musculus* |
| Genotyping | f1: GGCCTCAAACAAGTTATGCAG  f2: TAAACGATCTTCCCGTTGGCC  r: CGATTAGGTAACTCTGGTTGTC | *Mus musculus* |

**Table S3. The antibodies and application**

| Antibodies | Catalogue | Company | Application |
| --- | --- | --- | --- |
| PRDX1 | 15816-1-AP | Proteintech | WB/IHC |
| GLUT-1 | 21829-1-AP | Proteintech | WB/IHC |
| PD-L1 | 66248-1-Ig | Proteintech | WB |
| HIF-1α | sc-53546 | Santa Cruz | WB |
| Ubiquitin | L020707 | PTM Bio | Co-IP/WB |
| Phospho-STAT3 (Tyr705) | 9145 | Cell Signaling Technology | WB |
| STAT3 | 12640 | Cell Signaling Technology | WB |
| Phospho-NF-κB (Ser536) | 3033 | Cell Signaling Technology | WB |
| NF-κB | 8242 | Cell Signaling Technology | WB |
| Phospho-STAT1 (Tyr701) | TP56498 | Abmart | WB |
| STAT1 | T55227 | Abmart | WB |
| Phospho-AKT | 4060 | Cell Signaling Technology | WB |
| AKT | 9272 | Cell Signaling Technology | WB |
| HK1 | 2024 | Cell Signaling Technology | WB |
| PKM1/2 | 3190 | Cell Signaling Technology | WB |
| PKM2 | 4053 | Cell Signaling Technology | WB |
| LDHA | 3582 | Cell Signaling Technology | WB |
| PFKP | 8164 | Cell Signaling Technology | WB |
| HA tag | 51064-2-AP | Proteintech | Co-IP/WB |
| FLAG tag | 20543-1-AP | Proteintech | Co-IP/WB |
| Normal Rabbit IgG | 2729 | Cell Signaling Technology | Co-IP |
| GAPDH | 60004-1-Ig | Proteintech | WB |
| β-actin | 66009-1-Ig | Proteintech | WB |
| CD206 | ab64693 | Abcam | IHC |
| CD4 | GB13064-2 | Servicebio | IHC |
| CD8 | GB114196 | Servicebio | IHC |
| CD163 | GB113751 | Servicebio | IHC |

**Table S4. Antibodies for identification of the immunocytes using flow cytometry**

| **Antibodies** | **label** | **Immune cell types** | **Species** | **Catalogue** |
| --- | --- | --- | --- | --- |
| CD3 | Brilliant Violet 510 | T lymphocytes | mouse | Biolegend 100234 |
| CD4 | Alexa Fluor 647 | T lymphocytes | mouse | Biolegend 100424 |
| CD8a | Alexa Fluor 700 | T lymphocytes | mouse,human | Biolegend 100730 |
| CD11b | FITC | macrophages | mouse | Biolegend 101206 |
| F4/80 | PerCP/Cyanine5.5 | macrophages | mouse | Biolegend 123127 |
| CD86 | PE | macrophages | mouse | Biolegend 105008 |
| CD206 | APC | macrophages | mouse | Biolegend 141707 |
| IFN-γ | Alexa Fluor 488 | T lymphocytes | mouse | Biolegend 505815 |
| TNF-α | PE | T lymphocytes | mouse | Biolegend 506305 |
| GzmB | PE/Cyanine7 | T lymphocytes | mouse | Biolegend 372213 |
| PD-1 | Brilliant Violet 421 | T lymphocytes | mouse | Biolegend 135217 |

**Table S5. The significantly upregulated genes in CRC tissues of PRDX1-KO v.s. WT mice**

| Gene Symbol | Log FC | P value (PRDX1-KO_AD /WT_AD) | | Regulation |
| --- | --- | --- | --- | --- |
| Elovl3 | 21.86171206 | 2.22E-08 | Up | |
| LOC115490200 | 7.14274526 | 2.23E-18 | Up | |
| Retn | 6.68077293 | 4.26E-04 | Up | |
| Gm21451 | 6.265990674 | 6.19E-10 | Up | |
| H4c17 | 6.23898842 | 3.36E-05 | Up | |
| Mb | 5.691657986 | 1.35E-04 | Up | |
| Car3 | 5.258954729 | 6.30E-09 | Up | |
| Myl1 | 4.544313064 | 1.46E-04 | Up | |
| Shisa3 | 4.276200922 | 2.77E-05 | Up | |
| Cacna1i | 3.18365851 | 3.94E-04 | Up | |
| Serpina1e | 3.094338229 | 1.50E-04 | Up | |
| Frem1 | 3.087642731 | 1.40E-04 | Up | |
| Plac9a | 3.057824844 | 9.78E-08 | Up | |
| Spib | 2.845405572 | 3.22E-04 | Up | |
| Hapln1 | 2.636318039 | 2.58E-04 | Up | |
| Pax5 | 2.566037982 | 4.37E-04 | Up | |
| Cr2 | 2.564044943 | 1.24E-04 | Up | |
| Ms4a1 | 2.522084616 | 4.76E-04 | Up | |
| H2-Eb2 | 2.509741936 | 4.67E-04 | Up | |
| Xlr4a | 2.429335092 | 4.06E-04 | Up | |
| Cd22 | 2.418227569 | 2.72E-04 | Up | |
| Cd19 | 2.394886806 | 2.29E-04 | Up | |
| Fcrla | 2.278532077 | 2.87E-04 | Up | |
| Cphx1 | 2.261820934 | 1.66E-09 | Up | |
| Ccl21d | 1.888763237 | 1.98E-08 | Up | |
| Gm10591 | 1.885618882 | 2.38E-08 | Up | |
| Gm13304 | 1.885618882 | 2.38E-08 | Up | |
| Islr2 | 1.86465 | 1.45E-04 | Up | |
| Ccl21a | 1.75325957 | 2.08E-15 | Up | |
| Ifi27l2a | 1.699879599 | 8.80E-06 | Up | |
| Sell | 1.643582597 | 1.84E-05 | Up | |
| Bst1 | 1.641618812 | 2.34E-04 | Up | |
| Tmem254a | 1.563074796 | 2.45E-07 | Up | |
| Slc15a2 | 1.484020871 | 4.35E-04 | Up | |
| Gfra2 | 1.454067912 | 6.11E-06 | Up | |
| Lyve1 | 1.441706361 | 2.66E-07 | Up | |
| Mmrn1 | 1.423860369 | 2.76E-06 | Up | |
| Cyp26b1 | 1.325552113 | 3.46E-04 | Up | |
| Reln | 1.246174596 | 4.32E-04 | Up | |
| Srd5a2 | 1.213006404 | 3.56E-06 | Up | |
| Igfbp6 | 1.184278688 | 3.45E-04 | Up | |
| Flt4 | 1.143511024 | 2.21E-06 | Up | |
| Pyy | 1.056732424 | 3.35E-04 | Up | |
| Stmn2 | 1.048301682 | 1.37E-04 | Up | |
| 2610528A11Rik | 0.996785728 | 2.79E-04 | Up | |
| Calcrl | 0.98250775 | 4.11E-04 | Up | |
| Bin1 | 0.948396559 | 1.42E-04 | Up | |
| Entpd4b | 0.921759286 | 5.04E-06 | Up | |
| Cd34 | 0.858228067 | 1.29E-04 | Up | |
| Ptprm | 0.817051172 | 4.81E-04 | Up | |
| Ets1 | 0.689684925 | 6.63E-06 | Up | |
| Adam19 | 0.631886571 | 4.06E-04 | Up | |
| Tiparp | 0.614571115 | 4.90E-04 | Up | |

**Table S6. The significantly differential genes in RAW264.7^PRDX1-KD^ v.s. RAW264.7^CON-KD^ (listed in part)**

| Gene Symbol | Log FC | *P* value (PRDX1-KD /Control) | | Regulation |
| --- | --- | --- | --- | --- |
| RTL5 | -10.95821 | 0 | Down | |
| C2CD6 | -10.74695 | 5.11E-163 | Down | |
| PCDHGC3 | -10.24396 | 0 | Down | |
| CSF3 | -8.098616 | 0 | Down | |
| PRDX1 | -8.039661 | 0 | Down | |
| CXCL3 | -7.733562 | 0 | Down | |
| CXCL2 | -7.546006 | 0 | Down | |
| FABP4 | -5.98811 | 0 | Down | |
| Csf2 | -5.898581 | 3.45E-08 | Down | |
| IGF2R | -5.510227 | 0 | Down | |
| CXCR4 | -5.438022 | 8.56E-37 | Down | |
| Ccl7 | -5.265304 | 1.44E-25 | Down | |
| CCL2 | -4.222878 | 1.11E-227 | Down | |
| Il34 | -4.096108 | 0.009516 | Down | |
| Il17b | -4.082089 | 0.006757 | Down | |
| Ccl3 | -2.610332 | 0 | Down | |
| CCL9 | -2.538831 | 0 | Down | |
| Ccl4 | -2.280949 | 0 | Down | |
| RGS1 | -2.259865 | 0 | Down | |
| PLK2 | -2.100958 | 0 | Down | |
| PLAUR | -1.55928 | 1.28E-287 | Down | |
| MARCKSL1 | -1.350278 | 3.29E-315 | Down | |
| CXCL9 | 3.841549 | 8.48E-03 | Up | |
| CLEC12A | 2.88624 | 4.09E-276 | Up | |
| C3 | 2.448206 | 2.05E-18 | Up | |
| Cx3cr1 | 2.220534 | 1.33E-223 | Up | |
| STAT1 | 1.364742 | 3.59E-245 | Up | |
| JAK2 | 1.090045 | 5.90E-61 | Up | |

**Table S7. The levels of cytokines in conditioned medium of RAW264.7^PRDX1-KD^ v.s. RAW264.7^CON-KD^**

| Protein ID | AveExp.RAW264.7^CON-KD^ | AveExp.RAW264.7^PRDX1-KD^ | Log FC | P.Value | Regulated |
| --- | --- | --- | --- | --- | --- |
| IL-6 | 15.49017 | 12.85127 | -2.6388988 | 0.0420470 | Down |
| IL-9 | 11.96579 | 11.38734 | -0.5784472 | 0.0393365 | Down |
| TNF-α | 16.84502 | 17.77629 | 0.9312715 | 0.0272344 | Up |
| IL-1β | 10.99994 | 11.45250 | 0.4525600 | 0.0049284 | Up |
| IL-3 | 10.67738 | 11.15398 | 0.4766061 | 0.0130642 | Up |

**Table S8. The levels of cytokines in conditioned medium of BMDMs^PRDX1-KO^ v.s. BMDMs^WT^**

| Protein ID | AveExp.BMDMs^WT^ | AveExp.BMDMs^PRDX1-KO^ | Log FC | P.Value | Regulated |
| --- | --- | --- | --- | --- | --- |
| IL-5 | 10.84061 | 11.34435 | 0.50374 | 6.08E-06 | Up |
| IL-10 | 10.64949 | 11.27065 | 0.62116 | 5.26E-05 | Up |
| IFN-γ | 10.67298 | 11.16988 | 0.49689 | 8.74E-04 | Up |
| IL-4 | 9.83739 | 10.44382 | 0.60643 | 6.22E-04 | Up |
| IL-13 | 11.86079 | 10.56723 | -1.29356 | 9.27E-04 | Down |
| CXCL1 | 9.75919 | 10.45458 | 0.69539 | 7.64E-04 | Up |
| VEGF | 12.44264 | 13.14355 | 0.70092 | 4.22E-04 | Up |
| IL-2 | 10.79423 | 11.28051 | 0.48628 | 5.70E-03 | Up |
| IL-1β | 10.60259 | 11.00944 | 0.40685 | 8.39E-03 | Up |
| IL-12 | 11.33077 | 11.62495 | 0.29417 | 2.43E-02 | Up |
